# Integrated single cell and bulk sequencing analysis identifies tumor reactive CXCR6^+^ CD8 T cells as a predictor of immune infiltration and immunotherapy outcomes in hepatocellular carcinoma

**DOI:** 10.3389/fonc.2023.1099385

**Published:** 2023-08-01

**Authors:** Xiaogang Li, Zheng Gao, Jiafeng Chen, Shanru Feng, Xuanming Luo, Yinghong Shi, Zheng Tang, Weiren Liu, Xin Zhang, Ao Huang, Qiang Gao, Aiwu Ke, Jian Zhou, Jia Fan, Xiutao Fu, Zhenbin Ding

**Affiliations:** ^1^ Department of Liver Surgery and Transplantation, Liver Cancer Institute, Zhongshan Hospital, Fudan University, Shanghai, China; ^2^ Key Laboratory of Carcinogenesis and Cancer Invasion, Chinese Ministry of Education, Shanghai, China; ^3^ Shanghai Xuhui Central Hospital, Zhongshan-Xuhui Hospital, Fudan University, Shanghai, China

**Keywords:** hepatocellular carcinoma, immune infiltration, single cell sequencing technology, biomarker, tumor reactive T cells, immunotherapy

## Abstract

**Background:**

Various immune cell types in the tumor microenvironment (TME) of hepatocellular carcinoma (HCC) have been identified as important parameters associated with prognosis and responsiveness to immunotherapy. However, how various factors influence immune cell infiltration remains incompletely understood. Hence, we investigated the single cell multi-omics landscape of immune infiltration in HCC, particularly key gene and cell subsets that influence immune infiltration, thus potentially linking the immunotherapy response and immune cell infiltration.

**Methods:**

We grouped patients with HCC according to immune cell infiltration scores calculated by single sample gene set enrichment analysis (ssGSEA). Differential expression analysis, functional enrichment, clinical trait association, gene mutation analysis, tumor immune dysfunction and exclusion (TIDE) and prognostic model construction were used to investigate the immune infiltration landscape through multi-omics. Stepwise regression was further used to identify key genes regulating immune infiltration. Single cell analysis was performed to explore expression patterns of candidate genes and investigate associated cellular populations. Correlation analysis, ROC analysis, Immunotherapy cohorts were used to explore and confirm the role of key gene and cellular population in predicting immune infiltration state and immunotherapy response. Immunohistochemistry and multiplexed fluorescence staining were used to further validated our results.

**Results:**

Patients with HCC were clustered into high and low immune infiltration groups. Mutations of CTNNB1 and TTN were significantly associated with immune infiltration and altered enrichment of cell populations in the TME. TIDE analysis demonstrated that T cell dysfunction and the T cell exclusion score were elevated in the high and low infiltration groups, respectively. Six risk genes and five risk immune cell types were identified and used to construct risk scores and a nomogram model. CXCR6 and LTA, identified by stepwise regression, were highly associated with immune infiltration. Single cell analysis revealed that LTA was expressed primarily in tumor infiltrating T lymphocytes and partial B lymphocytes, whereas CXCR6 was enriched predominantly in T and NK cells. Notably, CXCR6^+^ CD8 T cells were characterized as tumor enriched cells that may be potential predictors of high immune infiltration and the immune-checkpoint blockade response, and may serve as therapeutic targets.

**Conclusion:**

We constructed a comprehensive single cell and multi-omics landscape of immune infiltration in HCC, and delineated key genes and cellular populations regulating immune infiltration and immunotherapy response, thus providing insights into the mechanisms of immune infiltration and future therapeutic control.

## Introduction

Hepatocellular carcinoma (HCC) is the third leading cause of cancer-associated death worldwide (1). Immune-checkpoint blockade (ICB) has revolutionized the approach to cancer therapy and has shown strong anti-tumor activity in a subset of patients with advanced HCC (2, 3). Various immune cells in the tumor microenvironment (TME) play important roles in patient prognosis and the immunotherapy response (4–8). Accumulating evidence indicates that several factors influence infiltration of immune cells in tumors, including somatic mutations, epigenetic modulation, microbial components and chemokines (9). Dissecting the tumor immune microenvironment (TIME) and immune infiltration status of HCC could help guide the clinical application of immunotherapy. However, how key gene regulators and cell populations modulate the immune cell composition within the TME have not been completely understood. The rapid development of single cell RNA sequencing (scRNA-seq) technologies has accelerated the identification of diverse cellular populations and phenotypic states within tumors, thus providing unprecedented opportunities for revealing gene expression patterns in individual cells and determining functional differences among clinical phenotypes, particularly regarding the immune cells that have infiltrated into tumors (10, 11). In addition, single cell analysis enables the effects of specific genes and cellular populations on tumor immune infiltration to be disentangled.

Here, we performed combined bulk sequencing and single cell RNA sequencing analysis in patients with HCC, and investigated their immune infiltration characteristics and potential regulators. We found that CTNNB1 and TTN mutations were significantly associated with immune infiltration. Patients in the high infiltration group showed enrichment in both tumor infiltrating effector and exhausted T cells, whereas those patients in low-infiltration group exhibited higher T cell exclusion scores, which were associated with poor prognosis. Furthermore, LTA and CXCR6 were identified as key genes influencing immune infiltration, which are expressed mainly on T cell/B cell, and T cell/NK cell populations, respectively. In particular, CXCR6^+^ CD8 cells were characterized as tissue resident and tumor reactive T cell populations, and were associated with high infiltration and responsiveness to ICB. This study revealed the single and multi-omics landscape of immune infiltration and key mediators influencing immune infiltration and immunotherapy response, thus providing insights into how immune cell infiltration in the TME occurs and may be therapeutically targeted in the future.

## Methods

### Datasets and gene list

In this investigation, HCC dataset (n=476) in The Cancer Genome Atlas (TCGA, RRID: SCR_003193) database (https://www.cancer.gov/about-nci/organization/ccg/research/structural-genomics/tcga) was used to identify high and low immune infiltration groups and obtain differentially expressed genes (DEGs). In addition to TCGA database, data from a Japan HCC dataset from the International Cancer Genome Consortium (ICGC, RRID : SCR_021722) (https://dcc.icgc.org/repositories) and the GSE25097 dataset from Gene Expression Omnibus (GEO, RRID : SCR_005012) (https://www.ncbi.nlm.nih.gov/geo/) were collected for further immune infiltration classification. After exclusion of combined HCC and cholangiocarcinoma, recurrent HCC and other rare HCC pathological types from the TCGA LIHC cohort, we selected 355 patients with HCC in TCGA cohort for downstream analysis.

Single cell sequencing data for 18 patients with HCC was procured from the dataset CNP0000650. Another single cell dataset of CD45+ cells isolated from tumors and four immune-relevant sites (adjacent liver, hepatic LNs, blood and ascites) of 16 treatment-naïve patients with liver cancer were obtained from GSE140228. Two bulk sequencing immunotherapy cohorts were enrolled for the validation of key genes in immunotherapy response including melanoma cohort (GSE91061) and IMvigor210 cohort. Four single cell immunotherapy cohorts were used to validate the role of key cellular populations in immunotherapy response, including primary liver cancer cohort (GSE125449),melanoma cohort 1 (GSE120575), melanoma cohort 2 (GSE115978) and basal cell carcinoma cohort (GSE123813).

**Figure 1:**
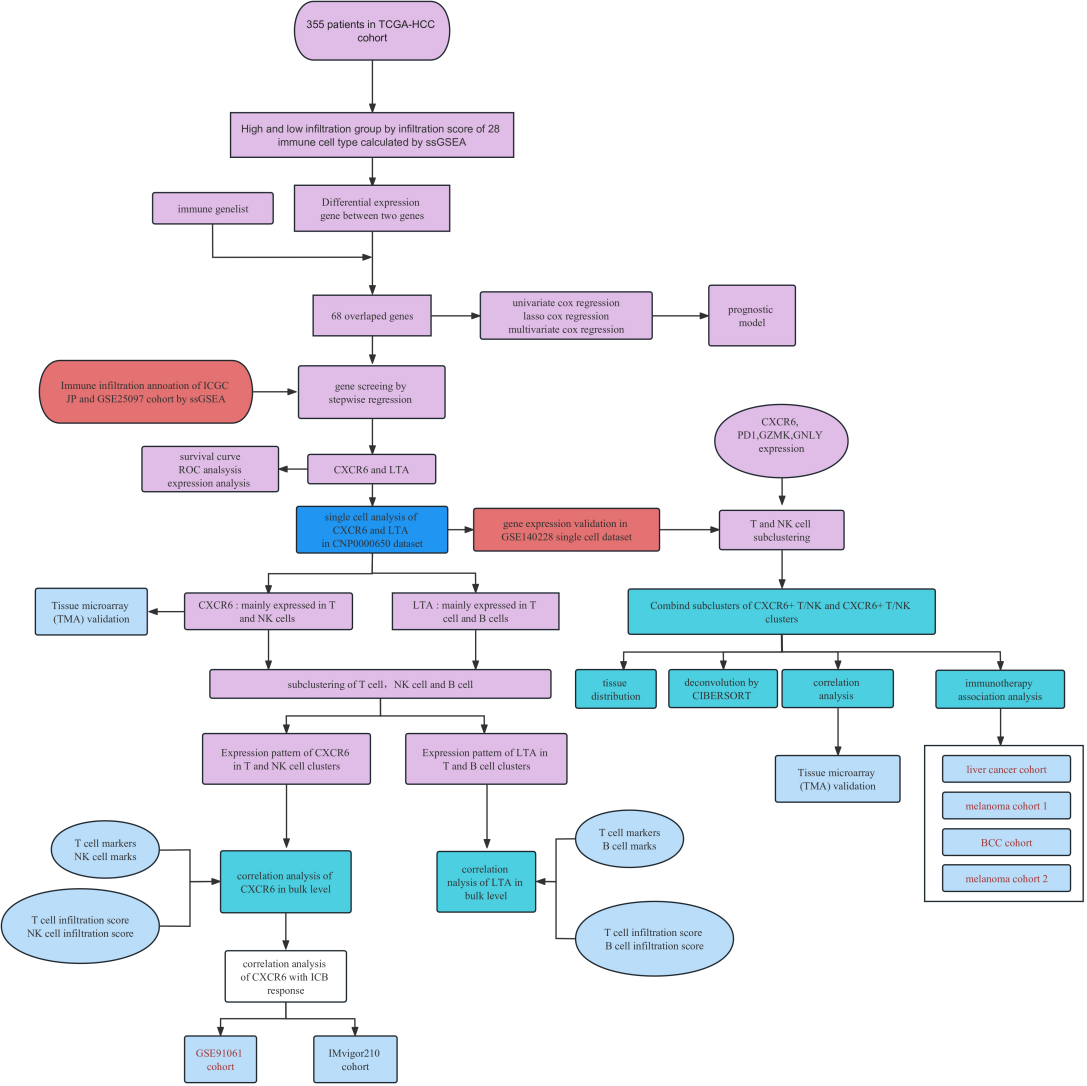
Work flow of study.

The immune meta gene lists for 28 immune cell types were downloaded from the Tumor Immune System Interactions Database (12) (http://cis.hku.hk/TISIDB/index.php). The immune associated gene lists were obtained from the Immport database. The workflow for this investigation is provided in **Figure 1**.

### Calculation of immune infiltration scores and differential expression analysis

In our analysis, single sample gene set enrichment analysis (ssGSEA) (13, 14) for immune infiltration annotation was performed to calculate the immune infiltration scores of 28 immune cell types, including activated CD4 T cells, activated CD8 T cells, activated dendritic cells, CD56 bright NK cells, central memory CD4 T cells, central memory CD8 T cells, effector memory CD4 T, effector memory CD8 T, NK cells, NKT cells, type 1 T helper cells, type 17 T helper cells, CD56 dim NK cells, immature dendritic cells, macrophages, myeloid-derived suppressor cells (MDSCs), neutrophils, plasmacytoid dendritic cells, regulatory T cells (Tregs), type 2 T helper cells, activated B cells, eosinophils, gamma delta T cells, immature B cells, mast cells, memory B cells, monocytes and T follicular helper cells (15). Clustering of patients into a high and low immune infiltration group was performed through hierarchical clustering (16). DEGs between patients with high versus low immune infiltration were analyzed with the DESeq2 package (RRID : SCR_015687) (https://bioconductor.org/packages/release/bioc/html/DESeq2.html)(17, 18).

### Functional enrichment analysis

For Gene Ontology (GO) and pathway enrichment, the DAVID database was used for GO analysis, including biological processes, cellular compartments and molecular functions(https://david.ncifcrf.gov/summary.jsp) (19). Protein domain and KEGG pathway analyses of DEGs between groups were also performed on data downloaded from DAVID database. The REACTOME database was also used for annotation of significantly differing pathways between groups (www.reactome.org) (20). Gene set enrichment analysis (GSEA, RRID : SCR_003199) (http://www.broadinstitute.org/gsea/) was used to demonstrate altered pathways, on the basis of DEGs between the high immune infiltration and low immune infiltration groups with the software GSEA v4.2.3 (SeqGSEA RRID : SCR_005724) (http://bioconductor.org/packages/devel/bioc/html/SeqGSEA.html) (Broad Institute, Inc., Massachusetts Institute of Technology, and Regents of the University of California) (13, 14). The annotation of changed pathways in this investigation was performed with the hallmarks gene set (version: 7.5.1) (21). Significantly enriched pathways were defined as those with P values < 0.05, |normalized enrichment scores (NES)| > 1 and false discovery rates < 0.25.

### Construction of a prognostic model

To focus on the role of immune regulation in predicting patient survival, we used overlapping genes between the immune associated genes and DEGs to determine predictive gene signatures and immune cell types. Univariate Cox regression analysis based on the Akaike information criterion (AIC) was performed to identify survival-associated genes with the “survival” R package (P < 0.05). Subsequently, on the basis of the prognosis-associated genes obtained from the above analysis, we used the “glmnet” package to conduct least absolute shrinkage and selection operator (LASSO) regression Cox analysis (simulation times = 1,000). Similarly, risk-cell types were identified with univariate Cox regression and LASSO regression Cox analysis, on the basis of the infiltration scores of 28 immune cell types. Furthermore, the risk-gene score and risk-cell type score were calculated by the risk gene coefficient multiplied by the gene expression value and risk-cell type coefficient multiplied by the infiltration score in each sample, respectively. Subsequently, patients in TCGA-HCC were divided into a high/low risk-gene group and high/low risk-cell type group on the basis of optimal cutoff points. Kaplan-Meier survival curves were plotted to confirm the association between the risk-score model and patients’ overall survival. Next, a time-dependent receiver operating characteristic (ROC) curve was drawn to evaluate the predictive ability of this model with the “timeROC” R package. To confirm the roles of the risk-gene score and risk-cell type score as independent prognostic factors, we conducted multivariate Cox regression analysis and assessed clinicopathological factors. To develop a nomogram to predict the 1-year, 2-year and 3-year overall survival probabilities of patients with HCC in TCGA cohort (22), we sequentially subjected three independent prognostic factors—T stage, risk-gene score and risk-cell type group—to a stepwise Cox regression model and constructed a nomogram.

### Gene mutation analysis

We used the Maftools package (https://genome.cshlp.org/content/28/11/1747) to analyze the somatic mutations in patients with HCC (23). Gene mutation summary plots were used to demonstrate the statistical results for gene mutations. Waterfall plots were used to depict and compare the top mutations and their mutation frequencies. On the basis of the selected top gene mutations, we grouped patients with HCC into mutant type and wild type, and explored the association between gene mutation and immune cell infiltration. Furthermore, we calculated tumor mutation burden (TMB) and explored its relationship with the ESTIMATE immune score and patients’ overall survival.

### Immunotherapy response prediction

Many factors can affect ICB (immune checkpoint blockade) response (24). Predicting tumor response to ICB accurately requires the deep understanding to the molecular mechanism of tumor immune escape from immune system. Recent research have demonstrated two distinct mechanisms of tumor immune escape, including T cell dysfunction and T cell exclusion (25, 26). T cell dysfunction means the dysfunctional state of T cells in tumor immune microenvironment. Although some tumors have a high infiltration level of cytotoxic T cells, these T cells tend to be in a dysfunctional state, which will affect ICB effectiveness (27). T cell exclusion means some immunosuppressive factors that might exclude T cells from tumor infiltrating, which leads to low T cell infiltration (27).

We used the computational framework named Tumor Immune Dysfunction and Exclusion (TIDE) to predict ICB response of tumor patients, which consist of T cell dysfunction score and T cell exclusion score (28). T cell dysfunction score are calculated by the expression level of genes that can interact with cytotoxic T lymphocyte (CTL) and inhibit T cell function. The gene signatures that represent T cell dysfunction include TGFB1, SOX10 and so on (28). On the other hand, T cell exclusion score is calculated by the gene signatures that preclude the tumor infiltration of T cells, which represent the three cell types reported to restrict T cell infiltration including cancer-associated fibroblasts (CAFs),myeloid-derived suppressor cells (MDSCs) and the M2 subtype of tumor associated macrophages (TAMs) (28).

The TIDE algorithm can predict patients’ responses to immunotherapy on the basis of RNA expression profiles (28). We used the TIDE website algorithm to evaluate patients’ immunotherapy responses according to the TIDE scores. We further compared the T cell dysfunction score and T cell exclusion score between groups to assess differences in immunotherapy response. Subsequently, correlation analyses among the ESTIMATE immune score, TIDE score, T cell dysfunction score and T cell exclusion score were used to validate the above results. Expression levels of immune checkpoint molecules such as PD-L1 are essential in predicting patients’ responses to immunotherapy (29). Hence, we examined the expression levels of classical immune checkpoint molecules in the high-infiltration and low-infiltration group to further depict the different immune landscapes of the two groups.

### Screening of genes highly associated with immune infiltration

Bulk sequencing in the HCC cohort, including the ICGC JP and GSE25097 cohorts, was performed to further compress immune associated genes to identify genes highly associated with immune infiltration status. The ssGSEA algorithm was used to calculate the immune infiltration score for each sample. Subsequently, stepwise regression of the gene expression and infiltration score was performed in the ICGC JP and GSE25097 cohorts. The log transformed TPM value of gene expression and mean value for 28 immune cell type infiltration scores in each sample were used for stepwise regression.

### Correlation analysis

The TIMER 2.0 web tool (http://timer.cistrome.org) (30) was used to correlate gene expression with immune cell infiltration scores, which included scores calculated with the CIBERSORT (31) and MCP-counter methods. Scores of TCGA-HCC sequencing data from other infiltration estimating methods were calculated with the immunedeconv package (32). Gene Expression Profiling Interactive Analysis (GEPIA, RRID : SCR_018294) (http://gepia.cancer-pku.cn) (33) was used for correlation analysis among CXCR6, LTA, immune cell markers and a series of immune regulators in bulk sequencing data of HCC in TCGA database.

### Single cell RNA sequencing data processing

We used the Scanpy (version:1.9.1) (34) package to perform unsupervised clustering of single cells by using the read count matrix as input. First, the read counts for each cell were divided by the total count for that cell and multiplied by the scaling factor (10,000), then natural-log transformed. Cells were filtered according to the criteria of original research. After quality control, we performed principal component analysis on the normalized expression matrix by using highly variable genes identified by the ‘‘pp.highly_variable_genes’’ function. After the results of principal component analysis were obtained, the appropriate principal components were selected for calculating neighbors and Leiden clustering with the specific resolution parameters. After clustering, we used tSNE to visualize the cell clusters. Finally, to detect cluster-specific expressed genes, we compared the clusters pairwise by using the Scanpy ‘‘tl.rank_genes_groups’’ function and Wilcoxon test. For cell population annotation, we used the signatures chosen in the original publication and previously reported markers. For the T cell cluster, signatures of CD3D, CD3E, IL7R and ITM2A were chosen for annotation. For the NK cell cluster, FCGR3A, CD160, GNLY and NKG7 were chosen for annotation. For the B cell cluster, CD19, CD27, CD79A and MS4A1 were chosen. For the myeloid cell cluster, LYZ, AIF1, C1QB and RNASE1 were chosen for annotation. For the endothelial cell cluster, signatures of PECAM1, IGFBP7, SPARC and SPARC1 were chosen for annotation. For the epithelial cell cluster, signatures of KRT19, FXYD2, EPCAM and DEFB1 were chosen for annotation. For the malignant cell cluster, APOC3, ALB, APOA1 and APOA2 were chosen for annotation. The expression levels of genes of interest were visualized with dot plots and feature plots. Cell annotation of subtypes was performed on the basis of the top expressed markers

### T cell and NK cell clustering in the GSE140228 dataset

For the clustering of the T and NK cell clusters, we selected the top 20 principal components with a resolution parameter equal to 1. Notably, in contrast to the most commonly used cell annotation strategy based on the top expressed genes of each subcluster, we divided T and NK cells into subclusters according to the relative expression levels of CXCR6, PDCD1, GZMK and GNLY after scaling, in which the mean expression in each cluster was > 0.4, and a > 25% fraction of cells in each cluster was considered to indicate positive expression.

### Deconvolution of bulk sequencing samples

The relative abundance of infiltrating immune cell types was estimated with CIBERSORT with a signature matrix containing 22 functionally defined immune subsets (LM22) in each tumor sample from the TCGA HCC cohort (31). To evaluate the relative abundance of the T and NK cell subsets identified herein, we first constructed a custom signature matrix with CIBERSORTx (31) from the scRNA-seq data of T and NK cell clusters derived from the GSE140228 cohort (summarized as log_2_ transformed transcripts per million) with Single Cell Input Options = 0. On the basis of the signature matrix, the T and NK cell composition of each tumor sample was deconvolved with S-mode batch correction.

### Independent immunotherapy cohort validation

To validated the role of CXCR6 in immunotherapy response, transcriptomic data of melanoma cohort (GSE91061) and metastatic urothelial cancer cohort (IMvigor210) were preprocessing and normalization respectively. Then, the expression level of CXCR6 in pre-treated and post-treated patients, as well as non-responders and responders, were compared by statistical tests.

To further explore the role of CXCR6^+^ T cell in immunotherapy response, especially CD8^+^CXCR6^+^ T cell, we analyzed three independent single cell sequencing cohort. Raw counts or TPM data were used for initial data format. We used Seurat (V4.2.0) to perform normalization, variable genes selection, gene expression scaling, dimensionality reduction, finding neighbors and clustering. Percentage of mitochondrial genes, UMI counts, cell cycle scores and heat shock gene scores were calculated and regressed. To remove batch effects between samples, we used harmony to integrate single cell data of each sample. For GSE120575 dataset, we clustered all CD45+ immune cells and used classical markers for annotation of main immune cell types, including T cell, B cell, plasma cell, monocyte, macrophage and dendrite. Then, T cells were subseted for downstream analysis. For GSE123813 and GSE115978 dataset, we directly used the T cell data provided in the literature for further analysis. The number of principle components used for dimensionality reduction and finding neighbors ranged from 15-30 in different dataset. Resolution used for clustering ranged from 0.8-1.2 to get the optimal clustering results.

T cell data in three datasets was analyzed by the similar pipeline mentioned above. To explore the role of CXCR6^+^ T cell in immunotherapy response, we firstly annotated different T cell subclusters as CD8^+^ T or CD4^+^ T according to the expression of CD8A, CD8B, CD4. Then, we annotated CD8^+^ T or CD4^+^ T as CD4^+^CXCR6^+^, CD4^+^CXCR6^-^, CD8^+^CXCR6^+^ and CD4^+^CXCR6^-^ when the scaled mean expression of CXCR6 in each cluster was > 0 and the fraction of cells expressing CXCR6 in each cluster > 25%.

### Immunohistochemistry

A tissue microarrays (TMA) consisting of 90 HCC tissues and their paired non-tumor normal tissues, was constructed as described previously (35). Briefly, all patients in the Zhongshan HCC cohort study received a histopathological diagnosis of HCC by two histopathologists after surgical resection. Representative areas without necrotic and hemorrhagic material were pre-marked in the paraffin blocks and two cores were taken from representative tumor tissue and adjacent normal liver tissue to construct TMA slides. Then, TMAs were stained to examine the expression of CD3 (abcam, ab16669, 1:200), CD20 (abcam, ab78237, 1:200), CD68 (abcam, ab213363, 1:1000), respectively. Briefly, after baking in a thermostat dryer at 60°C for an hour, TMA sections were deparaffinized with xylene and rehydrated. 3% (vol/vol) hydrogen peroxide was used to quench endogenous peroxidase activity for 10 minutes, followed by four 3-minute washes with double-distilled water. Subsequently, the slides were immersed in 0.1 mol/L Tris-HCl solution (pH 9.2) and heated in a microwave oven for 30 minutes. After four 3-minute washes with PBS and being pretreated with PBS containing goat serum albumin (CWBIO, 01380/34021)for 20 minutes, the sections were incubated in a humidified box at 4°C overnight with corresponding primary antibodies. After three 3-minute washes with PBS, the sections were incubated with a biotinylated second antibody (ZSBIO, PV-6001) for 60 minutes at 37°C, followed by another four 3-minute washes with PBS. The reaction products were visualized using diaminobenzidine (ZSBIO, ZLI-9018) for 3 minutes (the detailed time are dependent on the color of the actual microscope slide), and counterstained with hematoxylin for 2 minutes. Images were acquired under a light microscope with a 40× objective lens (Olympus, Japan).

CD3, CD20, CD68 are proteins expressing on cell membranes. Therefore, we measured the expression of CD3, CD20, CD68 in TMA sections using the number of positive cells, proportion of positive cells and histochemistry score (H-Score) that integrated percentages of positive cells and staining intensities into the formula: (0 × % negative) + (1 × % weak) + (2 × % moderate) + (3 × % strong), respectively (36–38). The counts of CD3^+^, CD20^+^ and CD68^+^ cells and corresponding proportion were measured by overall field from each tissue spot. Images were analyzed and quantified by HALO software (Indica Labs, Corrales, NM, USA) The threshold for positive or negative staining was based on the optical density of the staining: regions above the positivity threshold were scored according to the optical density threshold set in the module; weakly positive is shown in yellow and strongly positive in red. The optimal threshold were determined according to actual staining images.

### Multiplexed fluorescence staining

Multiplexed fluorescence staining was performed as previously described (39 {Song, 2022 #205)}. In brief, 4-μm FFPE TMAs sections were deparaffinized in xylene and then rehydrated in 100, 90, and 70% alcohol successively. Antigen unmasking was performed with a preheated epitope retrieval solution, endogenous peroxidase was inactivated by incubation in 3% H2O2 for 20 min. Next, the sections were pre-incubated with 10% normal goat serum and then incubated overnight with primary antibodies panel: CD4 antibody (CST, 48274, 1:100), CD8 antibody (CST, 55336, 1:300) and CXCR6 antibody (abcam, ab273116,1:1000). Next, TMA sections were incubated with the corresponding HRP-conjugated goat anti-rabbit second antibodies (ZSBIO, CA) for 10-30 mins at room temperature. The antigenic binding sites were visualized using the OPAL dye: Opal -650 (AKOYA),Opal −520 (AKOYA), Opal- 570 (AKOYA) for each antibody, respectively. Then, TMA sections were counterstained with DAPI (Sigma) for 3-5 mins. Similar to the data analysis of immunohistochemistry, Multiplexed fluorescence staining images were analyzed and quantified by HALO software (Indica Labs, Corrales, NM, USA) as well. For the convenience of downstream comparisons, the CD4^+^CXCR6^+^, CD4^+^CXCR6^-^, CD8^+^CXCR6^+^ and CD4^+^CXCR6^-^ were analyzed, respectively.

### Statistics

Survival analysis in this investigation was performed using R packages “survival” and “survminer” which were used to identify the best cutoff values for survival comparison between groups. Statistical significance in survival analysis was assessed with log-rank tests. The Pheatmap package was used to construct heat maps. A generalized linear model in R was used for prediction of the immune infiltration status, by using overlapping genes between the lists of immune genes and DEGs. A stepwise algorithm (backward) was then used to choose the appropriate model according to the AIC extracted from the previously fitted model (AIC= −2*log L + k* edf; L: likelihood; edf: equivalent degrees of freedom). ROC curves were examined with the package pROC. A P value < 0.05 was considered significant.

## Results

### Different prognosis outcomes between patients with HCC clustered into high versus low immune infiltration groups

**Figure 2:**
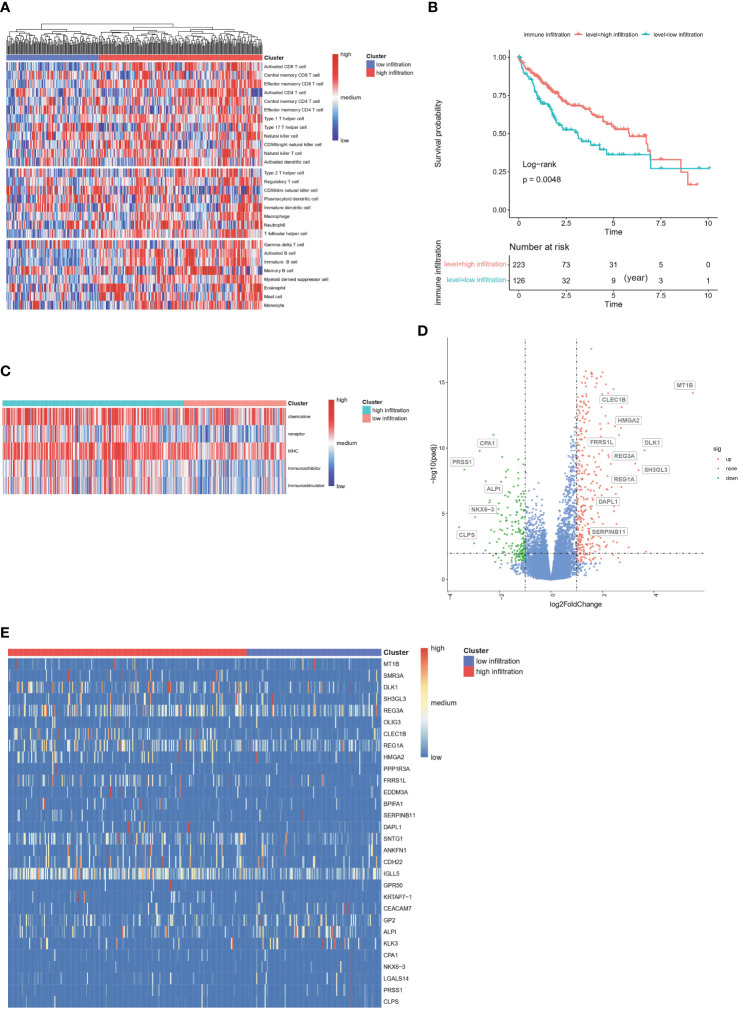
Construction of immune infiltration related clusters in TCGA-HCC cohort based on infiltration scores **(A)** Clustering of HCC patients according to immune infiltrated status calculated by ssGSEA algorithm. **(B)** Enrichment scores of chemokines, chemokine receptors, immune stimulators, immune inhibitors and MHC molecules between groups. **(C)** Kaplan-Meier survival analysis of high-immune and low-immune infiltrated HCC patients. **(D)** Volcano of differentially expressed genes between high- and low-immune infiltrated patients. **(E)** Heatmap plot of differentially expressed genes between high- and low-immune infiltrated patients.

In total, 355 patients in the TCGA-HCC cohort were included in downstream analysis. Using the immune gene list for 28 immune infiltrating cell populations, we generated scores for each immune cell type. After clustering patients with HCC according to the calculated infiltration scores, we found that immune status clearly differed between groups (**Figure 2A**, Additional file 1: **Table S1**). In addition, we used gene lists for immune stimulators, inhibitors, MHC molecules, chemokines and chemokine receptors to calculate the corresponding infiltration scores. In patients with high rather than low immune infiltration, the expression of these genes was much higher (**Figure 2B**). Moreover, survival analysis indicated that the prognosis of these patients was significantly better than that of patients with low immune infiltration (5-year survival rate 62% vs. 33%) (P =0.0048, **Figure 2C**).

### Immune functions and inflammatory signals are enriched in patients with HCC with high immune infiltration

**Figure 3:**
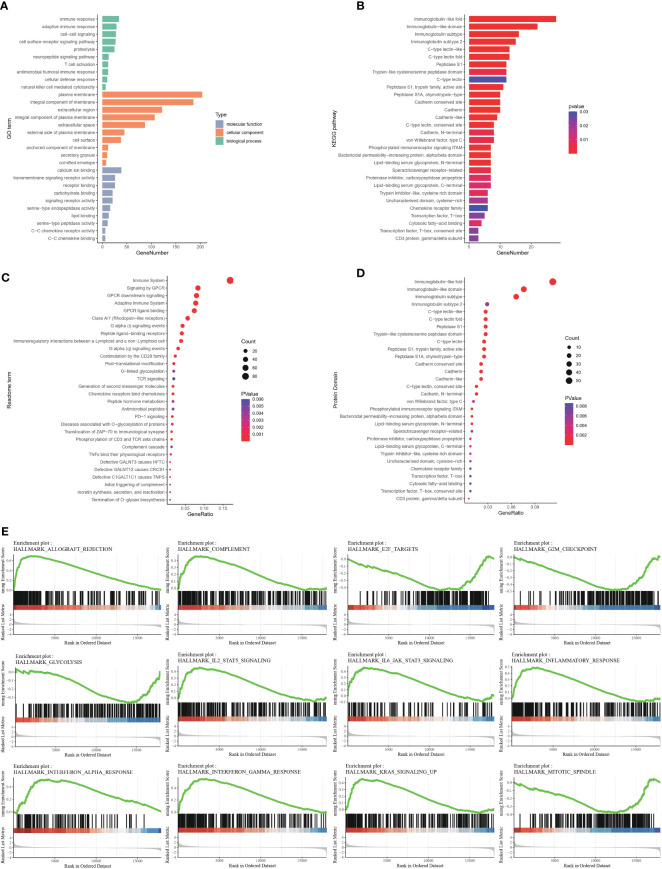
Functional enrichment of differentially expressed genes between high- and low-immune infiltration groups **(A)** Gene ontology enrichment of differentially expressed genes. **(B)** Pathway enrichment of differentially expressed genes between groups in KEGG database. **(C)** Pathway enrichment of differentially expressed genes between groups in REACTOME database. **(D)** Protein function enrichment of differentially expressed genes between groups. **(E)** Signatures of allograft rejection pathway, E2F targets, G2M checkpoints, complement pathway, glycolysis pathway, IL2-STAT5 pathway, IL6-Jak-STAT3 pathway, inflammatory response pathway, interferon-alpha response pathway, interferon-gamma response pathway, and KRAS signaling pathway and mitotic spindle were highly enriched in high immune infiltrated group.

DEGs between the high- and low-infiltration groups were analyzed. The total number of DEGs was 514, including 339 unregulated genes and 175 downregulated genes (Additional file 1: **Table S2**). Volcano and heatmap plots of DEGs in the high infiltration group compared with the low immune infiltration group were constructed (**Figures 2D, E**). GO gene set enrichment analysis revealed that the genes were involved in adaptive immune responses and T cell signaling (**Figure 3A**). KEGG enrichment analysis indicated that the related metabolic pathways included the T cell receptor signaling pathway, cytokine-cytokine receptor interaction and cell adhesion molecules (**Figure 3B**). Reactome pathway enrichment analysis indicated that the up-regulated gene set was involved in inflammatory signaling, immune stimulation and the PD1 axis (**Figure 3C**). Protein functional enrichment analysis indicated that most of the DEGs were immunoglobulins (**Figure 3D**). Further GSEA between high-infiltration group and low-infiltration group indicated high enrichment in complement signaling, IL2/STAT5 signaling, IL6/JAK/STAT3 signaling, inflammatory response signaling, interferon alpha signaling, interferon gamma signaling, KRAS signaling, glycolysis, the G2M checkpoint, E2F targets, allograft rejection and the mitotic spindle (**Figure 3E**).

### Clinical characteristics and tumor microenvironment traits in patients with HCC with high and low immune infiltration

**Figure 4:**
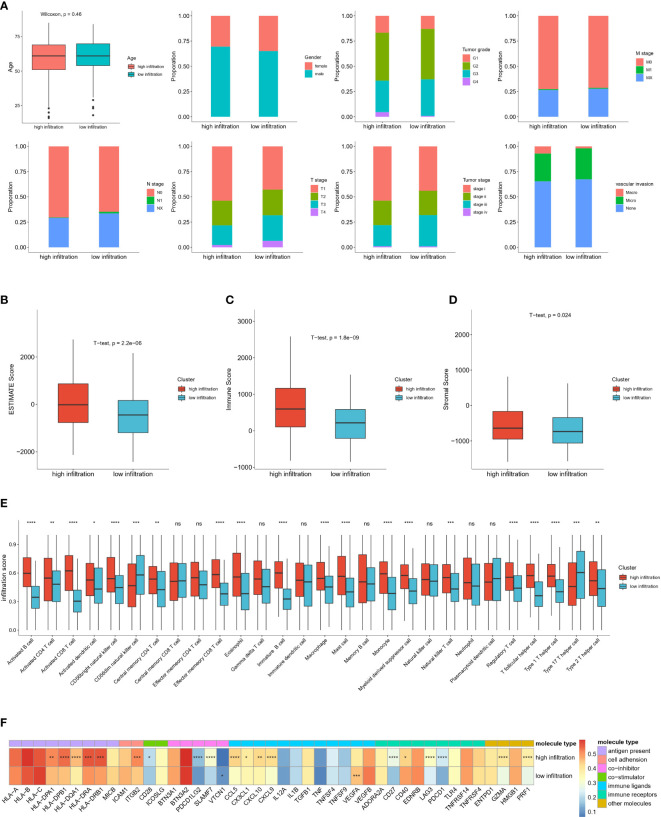
Clinical and immune microenvironment characterization of high immune infiltrated and low immune infiltrated clusters in TCGA-HCC cohort **(A)** Distribution of clinical characteristics of high- and low-immune infiltration groups. **(B)** Comparison of Estimate Score, Immune Score, Stromal Score, calculated by ESTIMTE algorithm between two groups. **(C)** Infiltration score of different immune cell types in high- and low-infiltration groups. **(D)** Expression level of immune associated molecules involved in antigen present, cell adhesion, co-stimulator, co-inhibitor, immune ligands, immune receptors and other immune molecules in high and low immune infiltration groups. **(E)** Boxplot shows the relative abundance of 28 immune cell types in high-infiltration and low-infiltration groups. Red and blue colors represent high-infiltration and low-infiltration groups, respectively. ns: no significance; *P<0.05; **P<0.01; ***P<0.001; and ****P<0.0001. **(F)** Heatmap show the average expression level and statistical significance of specific immune molecules in high-infiltration and low-infiltration group. Different colors represent different types of immune molecules. The color in each column represents the expression level of each immune molecules in this two groups. *P<0.05; **P<0.01; ***P<0.001; and ****P<0.0001.

We next explored the association between immune infiltration status and clinical traits. including age, sex, tumor histological grade, T stage, N stage, M stage, tumor stage and vascular invasion (**Figure 4A**). However, we observed no significant differences between the high-infiltration and low-infiltration group. Notably, stage I patients were more likely to be in the high infiltration group (53.81% vs 43.97%), whereas stage III patients were more likely to be in the low-infiltration (31.03% vs 20.95%). Similarly, the proportion of N0 stage patients with high immune infiltration was higher than that in patients with low immune infiltration (70.40% vs 64.8%). These results indicated a correlation between tumor grade and infiltration score. In addition, a higher ESTIMATE score, immune score and stromal score further verified the deep infiltration of immune cells and stroma cells (**Figures 4B–D**). Furthermore, 28 types of immune cells were analyzed separately in the two groups of patients (**Figure 4E**). Most immune cell types had high infiltration scores in the high infiltration group, particularly activated B cells and activated CD8 T cells. Interestingly, CD56 dim NK cells and type 17 helper cells exhibited higher infiltration scores in the low-infiltration group, thus suggesting that specific types of immune cells had higher enrichment levels in so-called cold tumors, and might play important roles in tumor immunity and be associated with low immune infiltration status. In support of the above findings, we observed greater enrichment in most immune molecules involved in antigen presentation, immune receptors, immune ligands, co-stimulators, and co-inhibitors in the high infiltration group than the low-infiltration group, with the exceptions of VTCN1 and VEGFA (**Figure 4F**).

### Gene mutations involved in HCC immune infiltration

**Figure 5:**
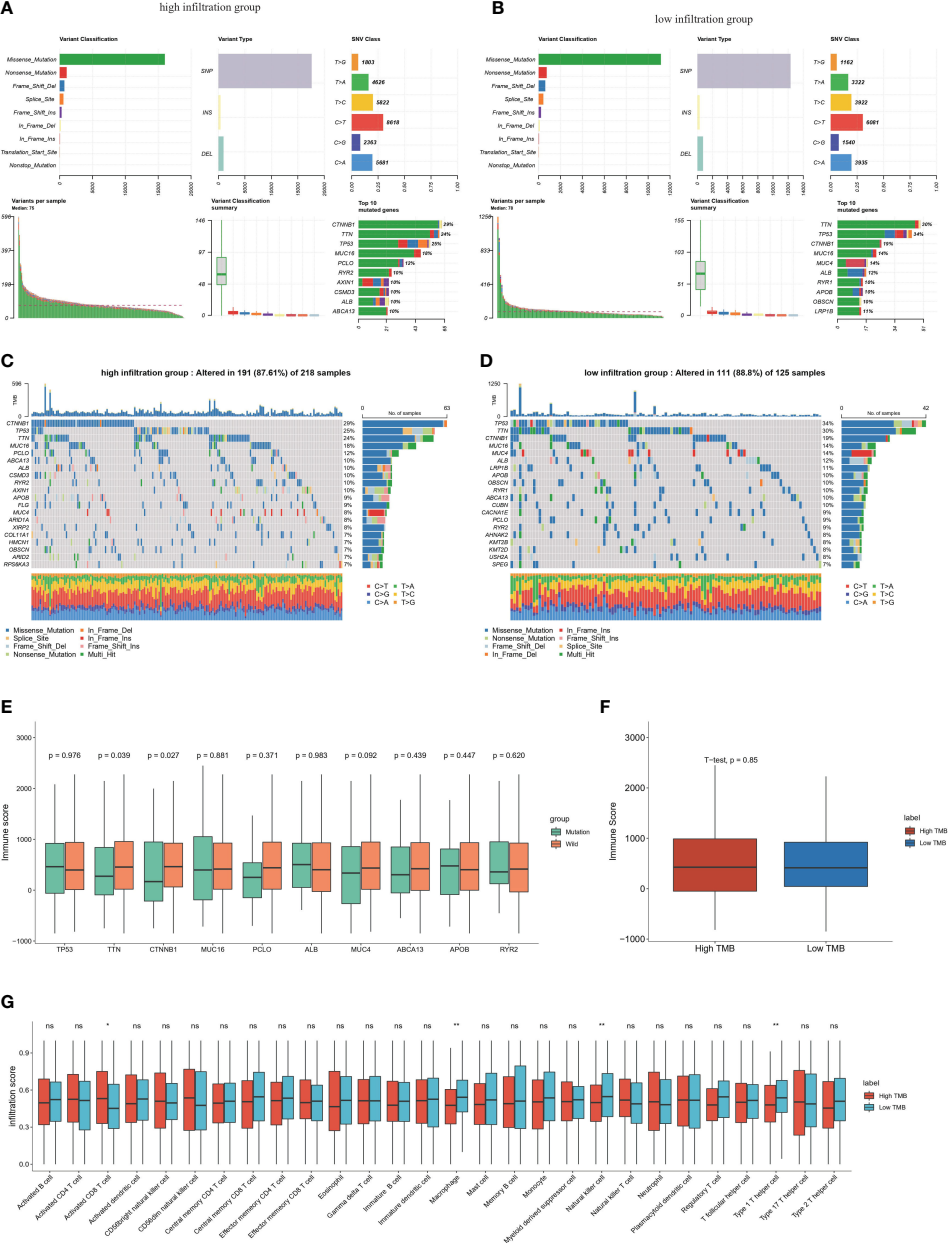
Genomic features of the high and low immune infiltrated clusters in TCGA-HCC cohort **(A)** Summary of somatic mutations in high-infiltration groups. **(B)** Summary of somatic mutations in low-infiltration groups. **(C)** Waterfall plot shows the top somatic mutations (top30) in high-infiltration group. **(D)** Waterfall plot shows the top somatic mutation (top30) in low-infiltration group. **(E)** The relationship between top somatic mutations and the ESTIMATE immune scores in high immune infiltrated group. Student’s t test was applied. **(F)** Boxplot of the relationship TMB and immune scores by ESTIMATE. **(G)** Infiltration score of different immune cell types calculated by ssGSEA algorithm in high and low TMB group. ns: no significance; *P<0.05; **P<0.01.

The mutation summary and plot of top mutations in the entire TCGA-HCC cohort indicated that the most common mutation type was missense mutation, and the top three mutations were TTN, TP53 and CTNNB1 (Additional file 1: **Figures S1A, B**). Significant differences were found in the top 20 gene mutations between the high-infiltration and low-infiltration group (**Figures 5A–D**). CTNNB1, a gene encoding a protein constituent of adherens junctions that is necessary for the creation and maintenance of epithelial cell layers (40), had significantly higher mutation rates in the high-infiltration group than the low-infiltration group (29% *vs* 19%). However, the most frequent mutation in the low-infiltration group was TP53, a classical mutation in HCC (41). These mutation differences indicated that several critical gene mutations determine the biological properties of tumor and the TME. Subsequently, the top six gene mutations, TP53, TTN, CTNNB1, MUC16, PCLO and ALB, were selected to explore the relationship between immune scores and gene mutations. Mutations of CTNNB1 and TTN significantly correlated with immune scores (**Figure 5E**), thus indicating that CTNNB1 and TTN are closely associated with immune cell infiltration. TMB is an important indication for evaluating tumor mutation and predicting the potential response to immunotherapy. We then calculated the TMB for each sample in the entire TCGA-HCC cohort, and analyzed the correlation between the immune score and TMB. However, no significant correlation between immune score and TMB was detected (**Figure 5F**). These results indicated a latent gap between tumor mutation burden and immune infiltration status, and further studies are necessary to determine the related mechanisms and specific immune cell types. In support of this finding, high TMB caused stronger enrichment in activated CD8 T cells, whereas low TMB was associated with higher infiltration scores for macrophages, NK cells and type 1 helper cells (**Figure 5G**), thus indicating that CD8 T cells are specifically activated in the context of high TMB. We next analyzed the immune cell infiltration scores of TTN and CTNNB1 mutant type or wild type groups, respectively. For TTN mutation, which was enriched in the low-infiltration group, we observed strong enrichment in central memory CD8 T cells and NK cells in the mutation group. (Additional file 1: **Figure S1C**). For CTNNB1 mutation, which was enriched in the high infiltration group, we observed significant enrichment in activated CD8 T cells, CD56 bright NK cells and effector memory CD8 T cells in the mutation group (Additional file 1: **Figure S1D**).

### Immunotherapy response prediction reveals distinct immune landscapes between the high-and low-infiltration groups

**Figure 6:**
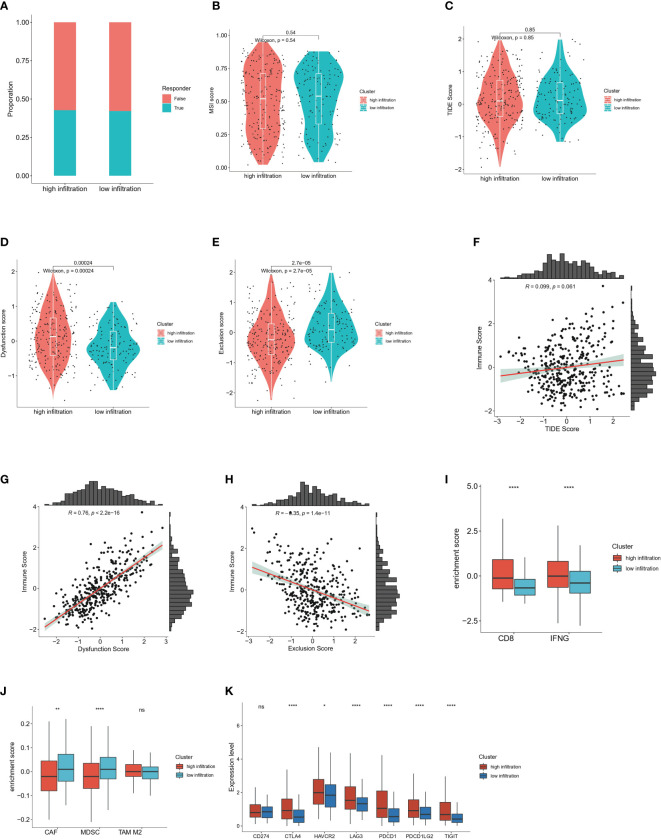
Immunotherapy response prediction of high and low immune infiltrated clusters **(A)** Proportion of responders and non-responders respectively in high and low infiltration group predicted by TIDE algorithm. **(B)** Comparison of microsatellite instability (MSI) score in high and low immune infiltration group predicted by TIDE algorithm. Wilcoxon test was applied. **(C)** Comparison of TIDE score in high and low immune infiltration group predicted by TIDE algorithm. Wilcoxon test was applied. **(D)** Comparison of T cell dysfunction score in high and low immune infiltration group predicted by TIDE algorithm. Wilcoxon test was applied. **(E)** Comparison of T cell exclusion score in high and low immune infiltration group predicted by TIDE algorithm. Wilcoxon test was applied. **(F)** Correlation analysis between the ESTIMATE immune score and the TIDE score. **(G)** Correlation analysis between the ESTIMATE immune score and T cell dysfunction scores. **(H)** Correlation analysis between the ESTIMATE immune score and T cell exclusion scores. **(I)** Enrichment score of CD8-associated molecules and IFNG pathway calculated by TIDE algorithm in high and low infiltration group. **(J)** Enrichment score of cancer-associated fibroblast (CAF), myeloid-derived suppressor cell (MDSC) and M2 type macrophages by TIDE algorithm in high and low infiltration group. **(K)** Expression levels of immune checkpoints in high and low immune infiltration group including CD274, CTLA-4, HAVCR2, LAG3, PDCD1, PDCD1LG2 and TIGIT. ns: no significance; *P<0.05; **P<0.01; and ****P<0.0001.

To further decipher the distinct immune landscapes between the high and low infiltration groups, we used the TIDE algorithm to predict the immunotherapy response of the two cohorts. Unexpectedly, the number of responders between groups did not significantly differ, thus suggesting that the overall immune infiltration status was unrelated to the immunotherapeutic response, and further indicating the complexity of the TIME (**Figure 6A**). In support of this finding, no significant differences in the microsatellite instability score and TIDE score were observed between groups (**Figures 6B, C**). Interestingly, the high-infiltration group had higher T cell dysfunction scores, whereas the low-infiltration group had higher T cell exclusion scores (**Figures 6D, E**). The T cell dysfunction score was defined as the infiltration level of inhibitory regulatory and exhausted T cells, whereas the T cell exclusion score represented the infiltration levels of factors hindering immune activation and effector T cell recruitment, including tumor associated macrophages (TAMs), cancer associated fibroblasts (CAFs) and MDSCs (28). In addition, correlation analysis among the TIDE score, T cell dysfunction score, T cell exclusion score and ESTIMATE immune score validated this result (**Figures 6F–H**). In support of this finding, higher enrichment levels of CD8 and the IFNG pathway were observed in the high-infiltration group, whereas greater enrichment of inhibitory cell populations including CAFs and MDSCs was observed (**Figures 6I, J**). The enrichment level of immune checkpoint molecules in tumor tissues indirectly represents the potential response to immunotherapy. Our analysis indicated that CTLA-4, HAVCR2, LAG3, PDCD1, PDCD1LG2 and TIGIT were significantly enriched in the high-infiltration group, but CD274, an inhibitory marker expressed mainly on surfaces of tumor cells and macrophages, was not significantly enriched(42) (**Figure 6K**).

### Construction of the prognostic immune, clinical and pathological model

**Figure 7:**
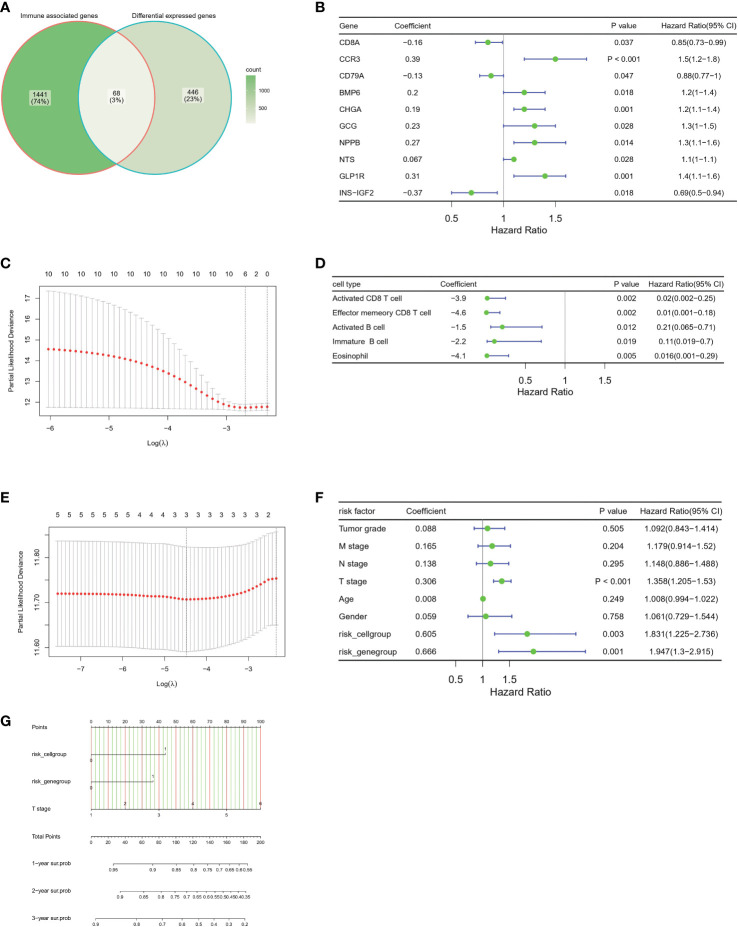
Construction of the prognostic immune, clinical and pathological model in the TCGA-HCC cohort **(A)** Venn plot show the overlap of immune associated genes and differential expressed genes between two groups. **(B)** Forest plots of the univariate Cox hazard model based on immune associated genes among DEGs. **(C)** Further screening of risk genes by partial likelihood deviance for LASSO coefficient profiles. The red dots represent the partial likelihood values, the gray lines represent the standard error (SE). **(D)** Forest plots of the univariate Cox hazard model of different immune cell type for overall survival. The 28 immune cell type infiltrated score are calculated by ssGSEA algorithm. **(E)** Further screening of risk immune cell types by partial likelihood deviance for LASSO coefficient profiles. The red dots represent the partial likelihood values, the gray lines represent the standard error (SE). **(F)** Forest plots of the multivariate COX hazard model of immune associated genes, immune cell types, clinical factors and pathological factors for overall survival. **(G)** Nomogram of individual survival risk prediction constructed by immune associated genes, immune cell type infiltration score, clinical factors and pathological factors.

To better understand the prognostic value of immune infiltration status, we chose 68 overlapping genes between DEGs and immune associated genes for the construction of prognostic model (**Figure 7A**, Additional file 1: **Table S3**). Ten gene signatures were retained after univariate Cox regression analysis, in which a P value < 0.05 indicated statistical significance (**Figure 7B**). Six genes, CD8A, CCR3, CD79A, CHGA, GLP1R and INS-IGF2, were selected as the best models through LASSO regression analysis and defined as risk genes (**Figure 7C**; survival curves of these six genes in Additional file 1: **Figures S2A-F**). Similarly, five immune cell types—activated CD8T cells, effector memory CD8 T cells, activated B cells, immune B cells and eosinophils (survival curves for these five cell types in Additional file 1: **Figures S2G-K**)—were defined as risk cell types after screening with univariate Cox regression analysis and LASSO regression analysis (**Figures 7D, E**). On the basis of the risk-gene score and risk-cell type score, patients with HCC in TCGA HCC cohort were divided into a high-risk group and low-risk group according to optimal cutoff points. Survival analysis showed that the high risk-gene or cell type groups had a significantly worse prognosis than low risk-gene or cell type groups (Additional file 1: **Figures S3A, B**). In addition, we investigated the survival prediction efficiency of the risk-gene score and risk-cell type score, both of which showed high sensitivity and specificity (Additional file 1: **Figures S3C, D**). To validate the independence of risk factors and construct a gene-cell type clinical predictive model, we used clinicopathologic factors including age, sex, tumor grade, T stage, N stage, and M stage combined with risk-gene group and risk-cell type group for multivariate Cox regression, and a p value of <0.05 was considered to indicate statistical significance (**Figure 7F**, Additional file 1: **Figure S3E**). T stage, risk-cell type group and risk-gene group were chosen for the construction of the nomogram. This nomogram incorporated clinical, pathological risk-gene and risk-cell type features and performed well in predicting patient survival probabilities, including 1-year survival, 2-year survival and 3-year survival (**Figure 7G**).

### The key role of CXCR6 and LTA in regulating immune infiltration

To investigate molecular predictors of immune infiltration status, we further calculated immune infiltration scores for the datasets ICGC JP and GSE25097, and divided patients with HCC into a high-infiltration group and low-infiltration group (clustering of high-infiltration and low-infiltration group in Additional file 1: **Tables S4**, **S5**, **Figures S4A, B**). We then used a backward stepwise regression model to compress the 68 gene set for prediction of immune infiltration status in two datasets (ICGC JP: infiltration score = 14.411 – 1.086 × CXCR6 – 0.809 × TNFRSF9 + 1.310 × LTA + 0.613 × TNFRSF17; GSE25097: infiltration score = 12.382 – 0.461×CD8A – 18.195 × LTA – 1.603×PCSK1 – 2.194×CD79A – 6.905 × NCR3 + 3.730 × STAB2 + 1.457 × CXCR6 + 16.779 × CXCR5 + 3.120 × NPPB) (**Table 1**).

**Table 1:** Stepwise regression model for compression of immune infiltration related genes, on the basis of the ICGC-JP and GSE25097 datasets.

Datasets		Estimate	Std. Error		t value	Pr(>|t|)	SS^#^
**ICGC-JP**	(Intercept)	14.41131	0.45892	31.403	< 2e-16		***
	LTA	1.31014	0.45212	2.898	0.00413		**
	CXCR6	-1.08554	0.35986	-3.017	0.00285		**
	TNFRSF17	0.61265	0.29522	2.075	0.03911		*
	TNFRSF9	-0.80870	0.38012	-2.128	0.03447		*
**GSE25097**	(Intercept)	12.3827	0.5651	21.913	< 2e-16		***
	CD8A	-0.4616	0.1679	-2.749	0.00640		**
	LTA	-18.1946	7.0282	-2.589	0.01018		*
	STAB2	3.7295	1.1505	3.242	0.00135		**
	PCSK1	-1.6033	0.5259	-3.049	0.00254		**
	CXCR6	1.4566	0.6075	2.398	0.01721		*
	CD79A	-2.1937	0.9657	-2.272	0.02394		*
	CXCR5	16.7794	5.6975	2.945	0.00353		**
	NPPB	3.1204	0.9082	3.436	0.00069		***
	NCR3	-6.9054	2.7445	-2.516	0.01248		*
	CTLA4	0.9458	0.3917	2.415	0.01646		*

^#^ statistical significance, signif. codes: 0 ‘***’ 0.001 ‘**’ 0.01 ‘*’ 0.05

**Figure 8:**
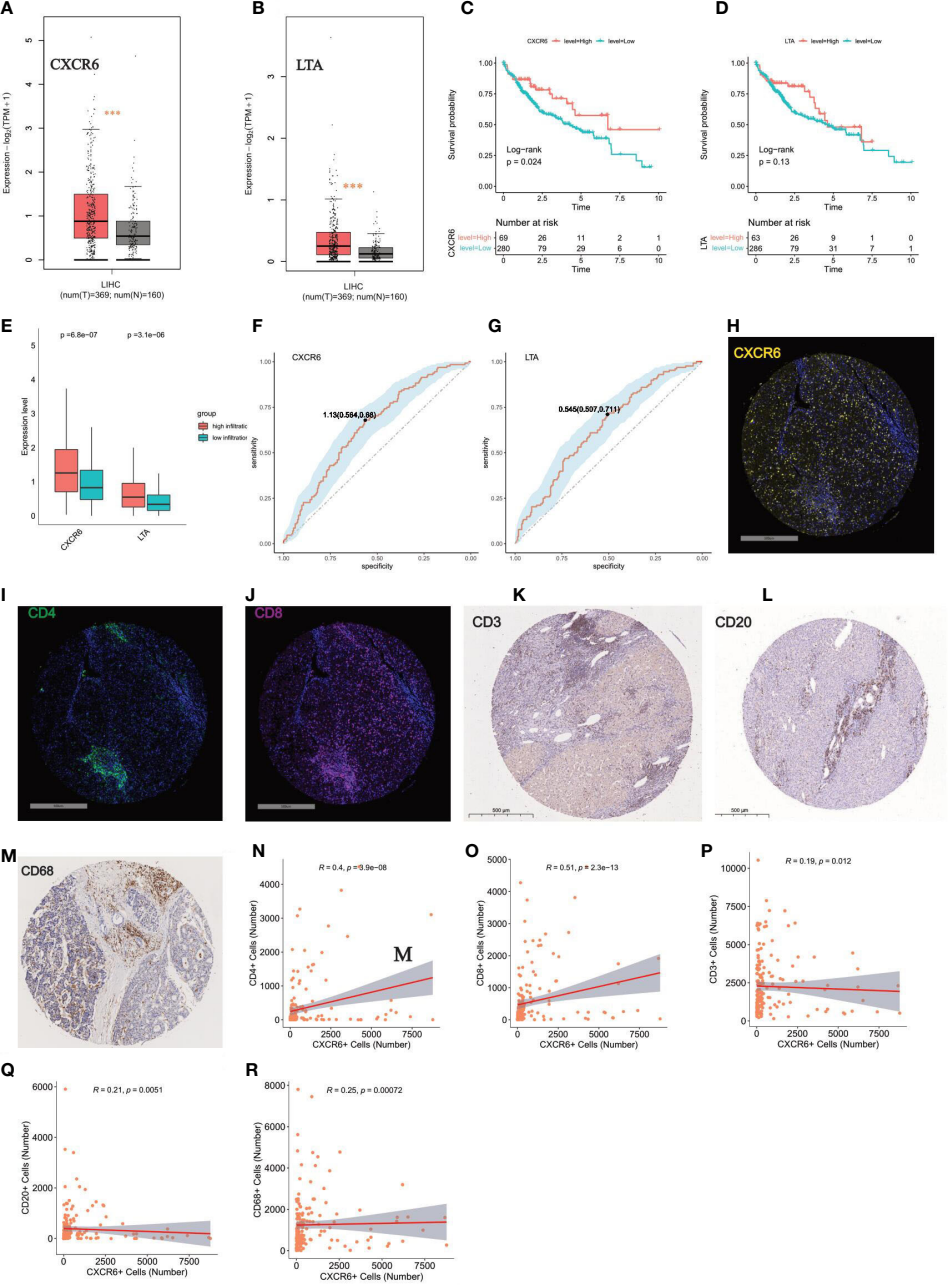
Key role of CXCR6 in regulating immune infiltration **(A, B)** Expression level analysis of CXCR6 and LTA in tumor tissue and normal liver by GEPIA tool, respectively. “***”: P < 0.001. **(C, D)** Kaplan-Meier survival analysis of CXCR6 and LTA in HCC patients, respectively. Log-rank test was used for statistical analysis. **(E)** Boxplot show the expression level of CXCR6 and LTA in high-infiltration and low-infiltration group. Wilcoxon test was applied. **(F, G)** ROC showed the sensitivity and specificity of CXCR6 and LTA in predicting high infiltration status, respectively. **(H–J)** Immunofluorescence (IF) staining of CXCR6, CD4, CD8 in our validation TMA cohort of 90 HCC cases, respectively. Representative images of positive staining are shown. Scale bar: 500 mm. **(K–M)** IHC staining of CD3, CD20, CD68 in our validation TMA cohort of 90 HCC cases. Representative images of positive staining are shown. Scale bar: 500 mm. **(N–R)** Correlation analysis between the expression level of CXCR6 with CD4, CD8, CD3, CD20, CD68 in our validation TMA cohort of 90 HCC cases. positive staining cells were counted and used for the correlation analysis.

Two overlapping genes, including LTA and CXCR6, were believed to be highly associated with immune infiltration status. Expression level analysis indicated that CXCR6 and LTA had higher expression in tumor tissues than normal tissues (**Figures 8A, B**). In addition, higher expression of CXCR6 indicated better survival, whereas LTA did not show statistical significance (**Figures 8C, D**).

To verify the roles of CXCR6 and LTA in immune infiltration status, we determined that the high-infiltration group, compared with the low-infiltration group, had significantly enriched expression of CXCR6 and LTA (**Figure 8E**). The ROC curve also demonstrated that LTA and CXCR6 were closely correlated with high immune infiltration status (**Figures 8F, G**). We next examined the correlation of CXCR6 with immune infiltration by immunochemical staining of our TMA sections (**Figures 8H–J**). High immune infiltration means more tumor infiltrating immune cells, so we stained CD3, CD4, CD8, CD20, CD68 to measure the immune infiltration status (**Figures 8K–M**). Interestingly, we found that CXCR6 have a strong correlation with CD3, CD4, CD8, CD20 and CD68 (P value < 0.05) (**Figures 8N–R**). Of all these markers, CXCR6 have the strongest correlation with CD8 and CD4 (**Figures 8N, O**). Taken together, we confirmed CXCR6 as an effective predictor of high immune infiltration from both transcriptomic level and protein level.

### Validation of the expression of LTA and CXCR6 at the single cell level

**Figure 9:**
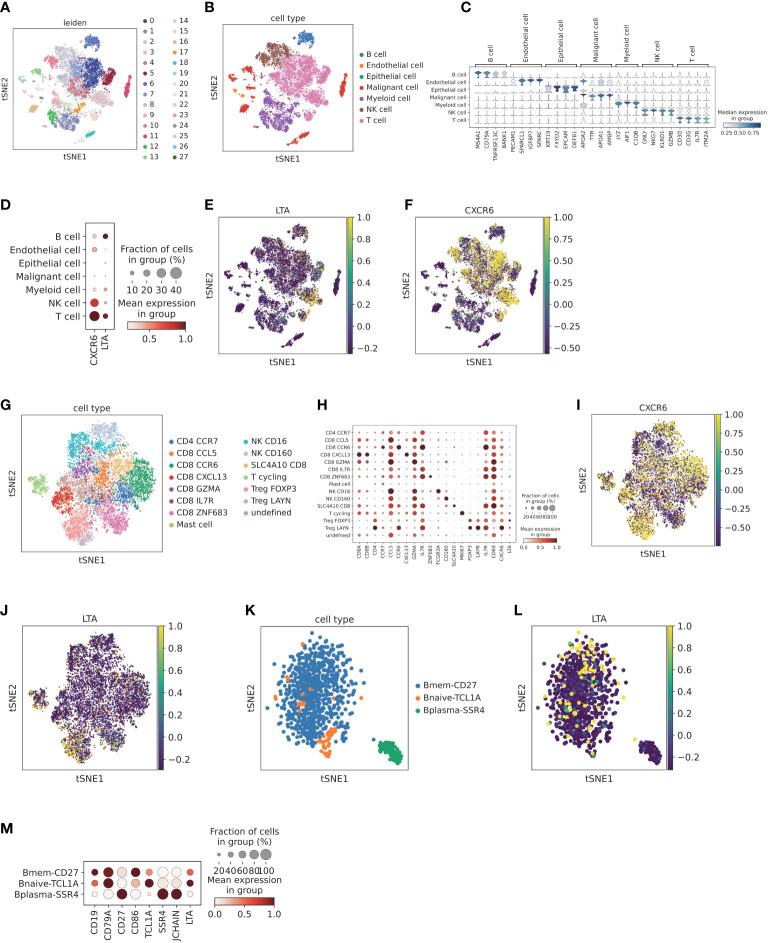
Validation of the expression of LTA and CXCR6 at the single cell level **(A)** Cell clusters for CNP0000650 dataset of 18 HCC patients. TSNE plot was colored by cluster. **(B)** Main cell type annotation visualized by TSNE. TSNE plot was colored by cell type. **(C)** Cell markers for cell type annotation. Violin plot was colored by the expression level of cell markers. **(D)** Dot plot of expression levels of LTA and CXCR6 in different cell clusters. Dot plot was colored by the expression level of cell markers and fraction of cells in each cell type. **(E)** Feature plot of expression levels LTA in different cell clusters, colored by expression level of LTA. **(F)** Feature plot of expression levels CXCR6 in different cell clusters, colored by the expression level of CXCR6. **(G)** Sub-clustering of T and NK cells according to top marker genes, visualized by TSNE. TSNE was colored by T and NK cell subtypes. **(H)** Cell markers for cell subtype annotation of T and NK cells, including CXCR6 and LTA. Dot plot was colored by the expression level of cell markers and fraction of cells in each cell subtype. **(I)** Feature plot of CXCR6 in T and NK cell populations, colored by the expression level of CXCR6. **(J)** Feature plot of LTA in T and NK cell populations, colored by the expression level of LTA. **(K)** Sub-clustering of B cells according to top marker genes, visualized by TSNE. TSNE was colored by B cell subtypes. **(L)** Feature plot of LTA in B cell populations, colored by the expression level of LTA. **(M)** Cell markers for cell subtype annotation of B cells, including LTA. Dot plot was colored by the expression level of cell markers and fraction of cells in each cell subtype.

After quality control, single cell HCC data were clustered into 28 subclusters (**Figure 9A**). A global UMAP plot and the marker genes of seven major annotated cell types are shown (**Figures 9B, C**). Expression analysis of LTA and CXCR6 in each major cell type was demonstrated with dot plots and feature plots. We found that LTA was expressed primarily in T cells and partially in B cells, whereas CXCR6 was expressed predominantly in T cells and NK cell subsets (**Figures 9D–F**).

Subsequently, we further explored the CXCR6 and LTA expression patterns by T cell and NK cell sub-clustering (**Figure 9G**). Among T cell and NK cell populations, CXCR6 was found to be expressed by Treg LAYN, Treg FOXP3, CD8 SLC4A10, CD8 CCR6, CD8 CXCL13 and NK CD160 (**Figures 9H, I**). In addition, LAYN+ regulatory T cells and CCR6+ CD8 T cells demonstrated the highest expression. Interestingly, CXCR6 was expressed mainly in CD160+ NK cells rather than CD16+ NK cells, which were predominantly distributed in adjacent normal tissue and represented tissue-resident memory NK cells (43). Previous reports have demonstrated that CXCR6 plays an important role in CXCR6^+^ NK cell recruitment to tumor tissue and participate in anti-tumor immunity (44). We next explored the expression pattern of LTA. After sub-clustering, Treg FOXP3, Treg LAYN and CD8 ZNF683 showed higher expression of LTA (**Figure 9J**). In B cell subpopulations, both memory B cells and naive B cells demonstrated high expression of LTA (**Figures 9K, L**). However, plasma cells scarcely expressed LTA (**Figure 9M**), thus potentially indicating that plasma cells are terminally differentiated and have no significant effect in promoting high immune infiltration.

We next investigated the correlation between LTA and CXCR6 expression and immune cell infiltration scores. We found that LTA was highly correlated with B cell infiltration and FOXP3^+^ regulatory T cell scores (Additional file 1: **Figures S5A–F**). Furthermore, correlation analysis showed that LTA was highly correlated with B cell markers (CD19, CD79A, BANK1 and MS4A1) and regulatory T cell markers (CD4, FOXP3, CD25 and CD39) in bulk sequencing data of TCGA HCC (P < 0.05; Additional file 1: **Figures S6A–L**). The expression of CXCR6 was also highly correlated with Treg, CD8+ T cell and NK cell infiltration scores (Additional file 1: **Figures S7A–J**). We then correlated CXCR6 with Treg cell markers (CD4, FOXP3, CD25 and CD39), CD8 T cell markers (CD8A, GZMB, TIM3 and PD1) and NK cell markers (CD160, NKG7 and GNLY) in the HCC dataset, all of which demonstrated high coefficients and statistical significance (Additional file 1: **Figures S8A–L**).

### CXCR6^+^ T cells characterized as tissue resident T cells and potential predictor of high infiltration status

**Figure 10:**
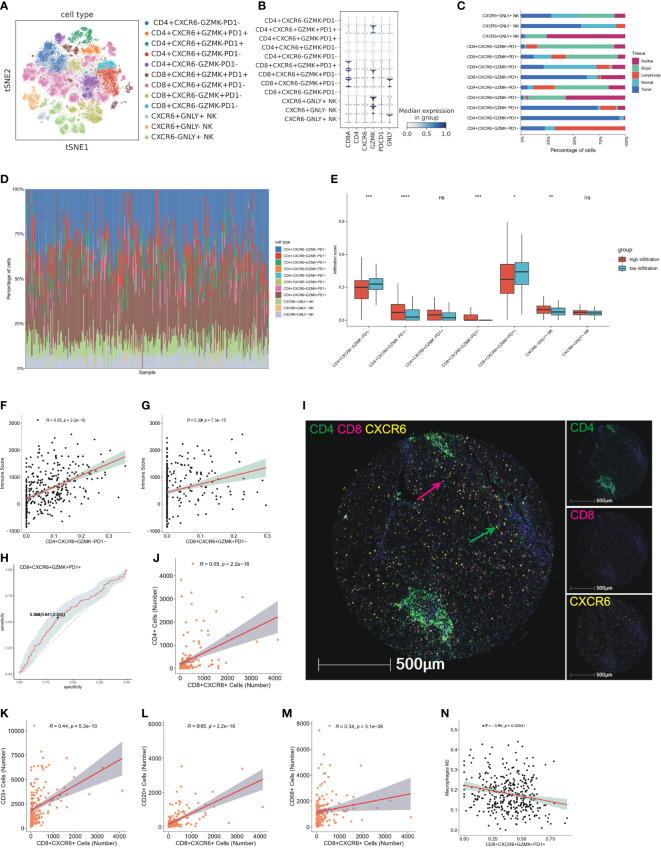
CXCR6^+^ T cells characterized as tissue resident T cells and potential predictor of high infiltration status **(A)** TSNE plot show further sub-clustering of T and NK cell populations according to relative expression level of CD4, CD8, CXCR6, GZMK, PDCD1 and GNLY, colored by cell subtype. **(B)** Violin plot showed the relative expression level of CD8A, CD4, CXCR6, GZMK, PDCD1 and GNLY in T and NK cell subsets. Expression level of marker genes are normalized to between 0 and 1. **(C)** Tissue distribution of 11 T and NK cell subsets in peripheral blood, ascites, adjacent liver tissue, tumor tissue and lymph node, colored by different tissue type. **(D)** Bar plot showed immune infiltration fraction of 11 T and NK subsets after cell deconvolution by CibersortX. Cell type signature are constructed by single cell matrix of T and NK cell populations. **(E)** Boxplot showed the infiltration score of T and NK subsets calculated by CibersortX in high-infiltration and low-infiltration group, colored by group. ns: no significance; *P<0.05; **P<0.01; ***P<0.001; and ****P<0.0001. **(F)** Representative correlation analysis between CXCR6^+^ subsets and immune score calculated by ESTIMATE. The scatter plot showed the correlation of CD4^+^CXCR6^+^GZMK^−^PD1^−^ and immune score. **(G)** Representative correlation analysis between CXCR6^+^ subsets and immune score calculated by ESTIMATE. The scatter plot showed the correlation of CD8^+^CXCR6^+^GZMK^+^PD1^−^ and immune score. **(H)** Representative ROC showed the sensitivity and specificity of CXCR6^+^ T cell in predicting high infiltration status. CD8^+^CXCR6^+^GZMK^+^PD1^+^ T cells are showed here. **(I)** Representative mIF images showing the distribution of CD4^+^ CXCR6^+^ and CD8^+^CXCR6^+^ T cells in HCC (n = 90) from our TMA cohort: CD4 (green), CD8 (red), CXCR6 (yellow) and DAPI (blue). Green arrows (CD4^+^CXCR6^+^), red arrows (CD8^+^CXCR6^+^). Scale bar, 500 μm. **(J–M)** Representative correlation analysis between CXCR6+ subsets and CD4, CD8, CD3, CD20, CD68 in our validation TMA cohort of 90 HCC cases. Positive staining cells were counted and used for the correlation analysis. Correlation analysis between CD8^+^CXCR6^+^ T cells and CD4, CD3, CD20 and CD68 were showed here. **(N)** Representative correlation analysis between CXCR6^+^ T subsets and infiltration score of M2 type macrophage. CD8^+^CXCR6^+^GZMK^+^PD1^+^ was showed here.

To further investigate the functional characteristics of highly CXCR6-expressing T cells/NK cells and explore the possible correlation between CXCR6 and cytotoxic markers or immune checkpoints, we first clustered another independent single cell HCC cohort of GSE140228 into six main cell types: T cells, NK cells, B cells, plasma cells, myeloid cells and mast cells (**Figures 10A, B**). We then validated the expression level of CXCR6 and LTA in different cell clusters. In support of our results in CNP0000650 dataset, CXCR6 and LTA exhibited similar expression patterns, in which CXCR6 was mainly expressed in T cell and NK cell while LTA have a preferential expression in T cell and B cell (Additional file 1: **Figures S9A-E**).

Next, we explored the characteristics of CXCR6^+^ T cells and CXCR6^+^ NK cells in the TIME. The T and NK cell populations were clustered into 11 cell subtypes according to the relative expression of CXCR6, GZMK, PDCD1 and GNLY and good clustering was observed (**Figures 10A, B**). Interestingly, analysis of the tissue distribution of CXCR6 associated cell populations indicated that CXCR6^+^ subsets were strongly enriched in tumor or normal tissue, particularly CD8^+^CXCR6^+^ T cells and CXCR6^+^ NK cells, whereas most CXCR6- subsets exhibited preferential enrichment in the peripheral blood (**Figure 10C**). CXCR6 have previously identified as a tissue resident marker and play an important role in regulating the settling down of T cells (45, 46). Therefore, we considered both CXCR6^+^ T cells and CXCR6^+^ NK cells as tissue resident and tissue specific immune cells. In support of our findings, we observed higher proportions of CXCR6^+^ T cell and CXCR6^+^ NK cell subsets than CXCR6^−^ cell subsets in high immune infiltration group by devolution analysis of bulk sequencing data, particularly CD8+CXCR6+ T cells (**Figures 10D, E**). Interestingly, more CD8+CXCR6+GZMK+PD1+ T cells were found in low-infiltration group, while CD8+CXCR6+GZMK+PD1- are dominantly enriched in high-infiltration group.

Previous bulk level correlation analysis has identified the association of CXCR6 and immune score calculated using ESTIMATE. We additionally found that CXCR6+ subsets positively correlated with the immune score, particularly for CD4^+^CXCR6^+^GZMK^-^PD1^-^ T cells and CD8^+^CXCR6^+^GZMK^+^PD1^-^ T cells, whereas CD4^+^CXCR6^-^GZMK^-^PD1^-^ exhibited a negative correlation with the immune score (**Figures 10F, G**; Additional file 1: **Figures S10A–F**). In addition, we investigated the predictive efficiency of CXCR6^+^ T cells for high immune infiltration status through ROC curve analysis, which indicated good performance in predicting high infiltration status (**Figure 10H**; Additional file 1: **Figures S11A–E**). We further validated the association of CXCR6^+^ T cell with immune infiltration by co-staining of CD4, CD8 and CXCR6 (**Figure 10I**). The results showed that both CD4^+^CXCR6^+^ and CD8^+^CXCR6^+^ T cells showed a strong correlation with CD4,CD8,CD3, CD20 and CD68 (**Figures 10J–M**, Additional file 1: **Figures S11F–Q**) and CD8^+^CXCR6^+^ T cells have the strongest correlation (**Figures 10J–M**), which suggested that CXCR6+ T cells are strong predictors for immune infiltration and might be therapeutically targeted in the future.

In addition to these analyses, to delineate potential factors impeding the infiltration of CXCR6^+^ T cells and potential differences of CXCR6^+^ and CXCR6^-^ subsets, we analyzed the correlations of tumor infiltrating M2 type macrophages and cell subsets and found that CD8^+^CXCR6^+^GZMK^+^PD1^+^ T cells were negatively correlated with M2 type macrophages, whereas CD4^+^CXCR6^-^GZMK^-^PD1^-^ cells were positively correlated with M2 type macrophages (**Figure 10N**, Additional file 1: **Figures S10G–I**).

### CXCR6^+^ CD8 T cells could be as a potential immunotherapy target

**Figure 11:**
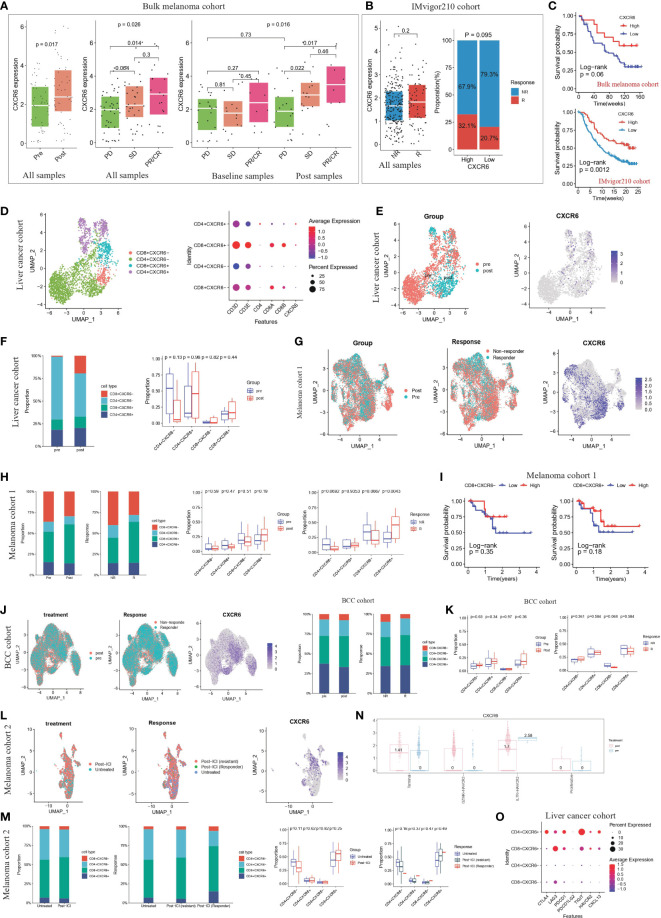
CXCR6^+^ CD8 T cells could be as a potential immunotherapy target **(A)** The expression level of CXCR6 from ICB baseline/post-treatment samples and responders/non-responders of bulk sequencing melanoma cohorts (47). The thick white line represents the median value, the bottom and top of the boxes are the 25th and 75th percentiles (interquartile range). Mann-Whitney test was used to calculate the significance between two groups. p < 0.05 were regarded as significant difference. PD: progressive disease; SD: stable disease; PR: partial response; CR: complete response. PR/CR were regarded as responders to ICB therapy. **(B)** The left panel represented expression level of CXCR6 from ICB responders/non-responders in IMvigor210 cohort (48). The thick white line represents the median value, the bottom and top of the boxes are the 25th and 75th percentiles (interquartile range). Mann-Whitney test was used to calculate the significance between two groups. The right panel represented the proportion of responders and non-responders in high-CXCR6 and low-CXCR6 group. Patients were grouped by the optimal cutoff point of CXCR6 expression. P < 0.05 were regarded as significant difference. NR: non-responders; R: responders. **(C)** Kaplan-Meier survival analysis of CXCR6 in bulk melanoma cohort and IMvigor210 cohort, respectively. Log-rank test was used for statistical analysis. Top panel represented the bulk melanoma cohort and bottom panel represented IMvigor210 cohort. **(D)** T cell re-clustering according to the expression level of CD4, CD8A, CD8B and CXCR6 in liver cancer cohort (49). Left panel represented the UMAP plot, colored by cell type. Right panel represented the dot plot of cell markers. **(E)** UMAP plots showing the clusters of T cells from liver cancer cohort (49) Cells were colored by the treatment of patients and expression level of CXCR6, respectively. **(F)** The proportion of CD4^+^CXCR6^+^, CD4^+^CXCR6^-^, CD8^+^CXCR6^+^ and CD4^+^CXCR6^-^ T cell from ICB baseline and post-treatment samples of liver cancer cohort (49). Left panel was colored by cell type; right panel was colored by the treatment. Mann-Whitney test was used to calculate the significance between two groups **(G)** UMAP plots showing the clusters of T cells from melanoma cohort 1 (50). Cells were colored by the treatment of patients, ICB response of patients and expression level of CXCR6, respectively. **(H)** The proportion of CD4^+^CXCR6^+^, CD4^+^CXCR6^-^, CD8^+^CXCR6^+^ and CD8^+^CXCR6^-^ T cell from ICB baseline/post-treatment samples and responders/non-responders of melanoma cohort 1 (50). Left panel was colored by cell type; right panel was colored by the treatment and response, respectively. Mann-Whitney test was used to calculate the significance between two groups. P < 0.05 were regarded as significant difference. **(I)** Kaplan-Meier survival analysis of CD8^+^CXCR6^-^ and CD8^+^CXCR6^+^ in melanoma cohort 1 (50). Log-rank test was used for statistical analysis. Left panel represented CD8^+^CXCR6^-^ and right panel represented CD8^+^CXCR6^+^. **(J)** UMAP plots showing the clusters of T cells from BCC cohort (51). Cells were colored by the treatment of patients, ICB response of patients and expression level of CXCR6, respectively. **(K)** The proportion of CD4^+^CXCR6^+^, CD4^+^CXCR6^-^, CD8^+^CXCR6^+^ and CD8^+^CXCR6^-^ T cell from ICB baseline/post-treatment samples and responders/non-responders of BCC cohort (51). Left panel was colored by cell type; right panel was colored by the treatment and response, respectively. Mann-Whitney test was used to calculate the significance between two groups. P < 0.05 were regarded as significant difference. **(L)** UMAP plots showing the clusters of T cells from melanoma cohort 2 (52). Cells were colored by the treatment of patients, ICB response of patients and expression level of CXCR6, respectively. **(M)** The proportion of CD4^+^CXCR6^+^, CD4^+^CXCR6^-^, CD8^+^CXCR6^+^ and CD8^+^CXCR6^-^ T cell from untreated/post-ICI samples and untreated/non-responders/responders of melanoma cohort 2 (52). Left panel was colored by cell type; right panel was colored by the treatment and response, respectively. Mann-Whitney test was used to calculate the significance between two groups. P < 0.05 were regarded as significant difference. **(N)** CXCR6 expression level in four CD8^+^ T cell subsets (terminal, GZMK^+^HAVCR2^-^, IL7R^+^ HAVCR2^-^ and proliferative) grouped by pre-treatment and post-treatment samples, by An interactive web server for analyzing and visualizing the scRNA-seq data (53). **(O)** Dot plot show the expression level of immune checkpoint molecules (CTLA4,LAG3,PDCD1,PDCD1LG2,TIGIT,HAVCR2,CXCL13) in CD4^+^CXCR6^+^, CD4^+^CXCR6^-^, CD8^+^CXCR6^+^ and CD8^+^CXCR6^-^ T cells from liver cancer cohort (49).

Because CXCR6 are closely related to high immune infiltration and better survival, we further investigate the correlation of CXCR6 expression with immunotherapy response. We studied this by two bulk sequencing patient cohorts who receive ICB therapy, including (1) bulk melanoma cohort, 73 melanoma patients; (2) IMvigor210 cohort, 348 patients with metastatic urothelial cancer. In bulk melanoma cohort, we found that the expression level of CXCR6 are elevated in post-treatment stage compared with baseline stage (**Figure 11A**). Consistently, responders exhibited higher expression level of CXCR6 compared with non-responders (**Figure 11A**). In addition, we examined the expression level of CXCR6 in responders and non-responders from baseline samples and post-treatment samples, respectively. We found that CXCR6 exhibited significant higher level in responders than non-responders, whether in baseline stage or post-treatment stage (**Figure 11A**). These results demonstrated that CXCR6 are positively correlated with immunotherapy response and might be play an important role in enhancing the immunotherapy effect. Similar to this results, IMvigor210 cohort also validated above results, in which CXCR6 show elevated expression in responders compared with non-responders (**Figure 11B**). Survival analysis showed a better survival of patients with higher CXCR6 expression (**Figure 11C**).

As mentioned above, CXCR6 expression are associated with better ICB response and longer survival. We wondered whether CXCR6^+^ T cells are correlated with clinical response to ICB and cancer patient outcome. We studied four single-cell sequencing patient cohorts that received ICB therapy, including (1) cohort 1, 19 primary liver cancer patients; (2) cohort 2, 32 metastatic melanoma patients; (3) cohort 4, 11 advanced basal cell carcinoma (BCC) patients; (4) cohort 3, 31 metastatic melanoma patients. In liver cancer cohort, after clustering T cell population into CD4^+^CXCR6^+^, CD4^+^CXCR6^-^, CD8^+^CXCR6^+^ and CD8^+^CXCR6^-^ T cell (**Figure 11D**; Additional file 1: **Figure S12A**), we found that CD8^+^CXCR6^+^ showed preferential enrichment in post-treatment samples compared with baseline samples (**Figures 11D, E**), which might suggest that CD8^+^CXCR6^+^ represented tumor-reactive T cells and CXCR6 were a potential marker for tumor-reactive T cells. Similar results were obtained from the other three cohort (**Figures 11G–M**; Additional file 1: **Figures S12B–G, I–K, M–O**) In addition, the proportion of CD8^+^CXCR6^+^ T cell were consistently higher in responders than non-responders (**Figures 11F, H, K, M**). In support of our findings, immunotherapy association analysis using an online database (53) revealed that the expression of CXCR6 is elevated in terminal differentiated T cell and GZMK^+^HAVCR^-^ T cell subsets after immune checkpoint blockade therapy, thus suggesting that CXCR6 positively correlates with the immunotherapy response (**Figure 11N**). In addition, previous study have demonstrated that CXCL13 could be a key marker in predicting the immunotherapy response, we also found that CXCR6^+^ T cells expressed higher level of CXCL13 and other immune checkpoint molecules, compared with CXCR6^-^ T cells, especially CD4^+^CXCR6^-^, CD8^+^CXCR6^+^ T cells (**Figure 11O**, Additional file 1: **Figures S12H, L,P**).

We next examined the relationship between the CXCR6^+^ T cell proportion and patient survival in melanoma cohort 1. We used the optimal cut off point calculated based on CXCR6^+^ T cell proportion of every patient to stratify patients into high and low groups. We found that compared with low CD8^+^CXCR6^+^ groups, high CD8^+^CXCR6^+^ group show longer overall survival (**Figure 11H**). High CD8^+^CXCR6^-^ T cell group also indicated longer survival (**Figure 11H**). However, high CD4+CXCR6+ T cell group suggested worse survival (Additional file 1: **Figures S12Q, R**). Taken together, these results strongly suggested that the expression levels of CXCR6 and proportion of CD8^+^CXCR6^+^ correlate to ICB response and are associated with patient outcomes.

## Discussion

By combining bulk sequencing and clinical information, we identified distinct immune cellular landscapes and clinical survival differences between the high-infiltration and low-infiltration group. We integrated transcriptomic sequencing data, gene mutation data, clinical information and single cell transcriptomic data for patients with HCC to reveal a detailed immune cellular landscape to support understanding of tumor infiltrating immune cells and key mediators that regulating immune infiltration. Notably, our findings highlighted CD8^+^CXCR6^+^ T cells as a potential predictor for immunotherapy response and target for cell-specific immunotherapy.

Previous attempts to identify immune infiltration groups have focused on molecular clustering to provide insights into personalized immunotherapy. High infiltration indicated better survival and a favorable response to immunotherapy, whereas low infiltration was associated with sparse tumor infiltrating immune cells. However, a low response rate to immunotherapy among patients with HCC remains a major obstacle to effective eradication or tumor cells. Therefore, measurements of baseline immune infiltration levels are insufficient to predict immunotherapy response and provide a reference for personalized immunotherapy design. In contrast, our analysis indicated that patients with high infiltration were characterized by enrichment in exhausted and regulatory T cells, whereas patients with low infiltration exhibited high enrichment in inhibitory cell populations, including TAMs, CAFs and MDSCs, which preclude effective recruitment and infiltration of cytotoxic T cells. In addition, CXCR6 and LTA expressed mainly in T cell subsets provided the highest power in discriminating immune infiltration in multiple HCC datasets, thus suggesting that CXCR6 and LTA may be effective biomarkers to represent immune infiltration status and guide clinical practice. Notably, tissue resident CXCR6^+^ T cells are highly tumor-reactive and correlate with immune infiltration and immunotherapy response, thus suggesting that CXCR6^+^ T cells may facilitate the rational design of T cell specific therapies (for example, immune checkpoint blockade therapy or engineered TCR-T cell therapy) for the treatment of human cancer.

Key gene mutations can alter immune infiltration and the immunotherapy response, thereby facilitating design of immunotherapy and combined therapeutics. Our analysis highlighted that CTNNB1 mutation was enriched in the high-infiltration group, while TTN mutation was enriched in low-infiltration group. Previous studies have demonstrated that mutation of CTNNB1 or TTN of tumor cells can affect the activation and recruitment of immune cells, thus regulating the infiltration of immune cells (54–56). CTNNB1 is a gene that encodes catenin beta-1 protein, beta-catenin is part of a complex of proteins that form adherens junctions, which are important for the establishment and maintenance of epithelial cell layers by regulating cell growth and adhesion between adjacent cells[12]. Mutant beta-catenin has been implicated in the pathogenesis of several cancers including melanoma, colorectal cancer, hepatocellular carcinoma, and ovarian cancer (57). TTN is gene that encodes a large abundant protein of striated muscle. Previous studies have mostly focused on a TTN mutation associated with muscle diseases (58). However, in recent years, increasing studies have demonstrated that TTN is implicated in the tumor mutation burden, chemotherapy response, immunotherapy response of solid tumors (59–61). The role of CTNNB1 and TTN in the regulation of immune cell infiltration are different. CTNNB1 mutation enriched in high-infiltration group was associated with stronger recruitment of activated/effector memory CD8 T subsets and less enrichment in monocytes, macrophages, type 2 helper cells and type 17 helper cells, thus suggesting that CTNNB1 mutation enhances anti-tumor immunity and facilitates immunotherapy. For TTN mutation enriched in low-infiltration group, there were strong enrichment of central memory CD8 T cell and nature killer cell in mutation group.

TMB has been regarded as a biomarker of the response to anti-PD1/anti-PDL1 therapies (62). However, many tumors with high TMB do not respond to immune checkpoint blockade therapy. In contrast, some responses occur in low-TMB tumors (63, 64), potentially because the TMB does not fully reflect the abundance of tumor reactive T cell populations, particularly cytotoxic T cells, given complex and dynamic interplay in TIME. In support of this assumption, in our analysis, we did not observe a significant association between TMB and overall immune infiltration. By investigating immune infiltration differences in specific immune cell subsets, we unexpectedly found that only activated CD8 T cells and NK cells were elevated in the high-TMB group, and were accompanied by diminished macrophage infiltration. Notably, CXCR6 and LTA, as calculated and validated in our model, were both expressed mainly in T cells and were powerful predictors of immune infiltration in patients with HCC in multiple datasets.

Hot tumors, defined by high immune infiltration, usually indicate a potential response to immunotherapy. However, many hot tumors do not respond to ICB therapy, probably because of accumulation of abundant dysfunctional T cell populations. In support of this hypothesis, enriched T cell dysfunction signatures in the high-infiltration group explained the low response rate of hot tumors to immunotherapy in clinical practice. Interestingly, high T cell exclusion scores in the low-infiltration group implicated that TAMs, CAFs and MDSCs were preferentially enriched in cold tumors and precluded cytotoxic T cell recruitment and activation. Therefore, we propose that patients with HCC may benefit from combined cell-specific therapeutics targeting tumor reactive T cells, macrophages and CAFs.

Risk gene groups were established based on the expression level of risk genes and the coefficient calculated by cox regression. Similarly, we divided patients into high or low-risk cell type groups according to the infiltration score of risk cell types. risk genes are derived from the differentially expressed genes (DEGs) between high-infiltration and low-infiltration group. Risk cell types were obtained from the 28 immune cell types that were used to group HCC patients into high-infiltration and low-infiltration group. Therefore, there are some kind of connection between the two types of groups. The expression of risk genes were related to the infiltration score of risk cell types, so there were some coincidence between risk-gene groups and risk-cell type groups. However, the infiltration score of one cell type are determined by multiple genes, and one single cell might affect the infiltration score of multiple cell types. In our study, we conducted multivariate cox regression analysis using clinicopathological factors, risk-gene score and risk-cell type score. Interestingly, we found that there were three independent prognostic factors: T stage, risk gene score and risk cell type group. Therefore, we could conclude that there were some connection between risk-gene groups and risk-cell type groups, while they could also be regarded as two independent prognostic factors.

In cancer studies, CXCR6 (C-X-C Motif Chemokine Receptor 6) plays an important role in regulating effector and regulatory T cell recruitment into tumor tissues, thus enabling targeted therapy that promotes local anti-tumor immunity (65–67). In addition, CXCR6 is upregulated during the conversion of memory stem-like into effector-like CTLs and represents an immune checkpoint determining the magnitudes and outcomes of anti-tumor immune responses (68). CXCR6 have been reported to play an key role in sustained tumor control mediated by CD8+ cytotoxic T cells (CTLs) (68, 69). In terms of HCC research, CXCR6 inhibits hepatocarcinogenesis by promoting NKT cell and CD4^+^ T cell dependent removal of senescent hepatocytes (70). In a preclinical cancer model, CXCR6 expression on infiltrating CD8+ T cells are significantly increased post anti-PD-1 treatment (69). Interestingly, the percentages of intra-tumoral CD8^+^ T cells and the therapeutic efficacy of PD-1 blockade were rapidly decreased in cxcr6^−/−^ mice (69). However, the role of CXCR6 and CXCR6^+^T cell in immunotherapy response have not been completely delineated at present. In bulk melanoma cohort and IMvigor210 cohort (metastatic urothelial cancer), we also found that anti-PD-1 therapy could significantly enhance the expression of CXCR6, especially in responders. Next, single-cell analysis of T cells from four patient cohorts that received ICB therapy revealed that the proportion of CD8^+^CXCR6^-^ are markedly elevated after anti-PD-1 therapy compared with CD8^+^CXCR6^-^, CD4^+^CXCR6^+^, CD4^+^CXCR6^-^ T cells, which suggested CD8^+^CXCR6^+^ are major tumor-reactive T cells to ICB therapy and the CXCR6 expression on infiltrating CD8^+^ T cells are significantly increased after ICB treatment. Taken together, CXCR6 and CD8^+^CXCR6^-^ T cells are effective predictor for immunotherapy response and potential target to enhance the efficiency of ICB therapy.

Previous have demonstrated that tissue resident CXCR6^+^ CD8 T cells are important components of tumor-infiltrating lymphocytes, which further evolve into GZMK^+^HAVCR2^-^ subsets and terminally differentiated T cells (71). Studies in human tumors and mouse models have demonstrated that both precursor and terminally differentiated T cells mediate tumor killing, and increased proportions of these subsets may contribute to favorable immunotherapy response (72, 73). In lung cancer, increased levels of precursor-like T cells in responsive tumors have been observed after treatment, thus suggesting that PD-1 blockade therapy preferentially blocks the differentiation from precursor to terminally differentiated cells after effective treatment (74, 75). In contrast to this observation, terminally differentiated cells account for most post-treatment responsive tumors in BCC, SCC or RCC (53). By online database, we found higher expression of CXCR6 in GZMK+HAVCR2- and terminally differentiated cells after immunotherapy, thus also validating that CXCR6 is an effective predictor of the immunotherapy response.

Our work has several limitations that highlight directions for future research. First, the sample size was limited, and larger cohorts of data are needed to generalize our findings and investigate how additional factors, such as tumor sites, tumor grade and disease subtypes, may be associated with CD8^+^CXCR6^+^ T cell subsets. Second, a detailed understanding of the distinct roles of CD8^+^CXCR6^+^ T cell subsets, including how TME factors regulate recruitment and how CD8^+^CXCR6^+^ T cells differentiated into other T cell subsets remains to be determined. Third, in addition to *in vitro* validation using our TMA cohorts, our in-silico analysis might require further experimental validation to facilitate clinical translation, including gain-and loss-of-function studies, and *in vivo* animal studies. Fourth, we explored the role of CXCR6 and CD8^+^CXCR6^+^ T cells in immunotherapy response using public ICB cohort. Further establishment of our own ICB cohort of HCC is important to fully understand the role of CXCR6 and CD8^+^CXCR6^+^ T cells.

## Conclusion

In summary, our studies revealed a comprehensive single cell multi-omics landscape of immune infiltration in HCC, and identified key genes and cell subsets influencing immune infiltration, thus providing insights into how immune infiltration might occur and be therapeutically controlled in the future.

## Data availability statement

The original contributions presented in the study are included in the article/**Supplementary Material**. Further inquiries can be directed to the corresponding authors.

## Author contributions

XGL, ZD, and XF conceived and designed the study. XGL, ZG, and JC provided research methods. XGL, ZG, JC, SF, XML, ZT, WL, XZ, AH, QG, and AK participated in implementation of the study. XGL performed the statistical analysis. XGL drafted and revised the manuscript. JZ, JF, YS, XF, and ZD and reviewed the whole manuscript, adapting the manuscript for final submission. All authors contributed to the article and approved the submitted version.

## Supplementary material

Supplementary Table S1 Immune infiltration score and Immune Infiltration Classification of Patients in Datasets of TCGA HCC cohort.

Supplementary Table S2 Differentially expressed genes between high-infiltration group and low-infiltration group in TCGA-LIHC group.

Supplementary Table S3 Immune infiltration score and Immune Infiltration Classification of Patients in Datasets of GSE25097 cohort.

Supplementary Table S4 Immune infiltration score and Immune Infiltration Classification of Patients in Datasets of ICGC JP cohort.

Supplementary Table S5 List of immune associated differentially expressed genes(IADEGs).

## References

[1] Sung H Ferlay J Siegel RL Laversanne M Soerjomataram I Jemal A. Global cancer statistics 2020: GLOBOCAN estimates of incidence and mortality worldwide for 36 cancers in 185 countries. CA Cancer J Clin (2021) 71:209–49. doi: 10.3322/caac.21660 33538338

[2] Sangro B Sarobe P Hervás-Stubbs S Melero I. Advances in immunotherapy for hepatocellular carcinoma. Nat Rev Gastroenterol Hepatol (2021) 18:525–43. doi: 10.1038/s41575-021-00438-0 PMC8042636 33850328

[3] Sperandio RC Pestana RC Miyamura BV Kaseb AO. Hepatocellular carcinoma immunotherapy. Annu Rev Med (2022) 73:267–78. doi: 10.1146/annurev-med-042220-021121 34606324

[4] Angelova M Charoentong P Hackl H Fischer ML Snajder R Krogsdam AM. Characterization of the immunophenotypes and antigenomes of colorectal cancers reveals distinct tumor escape mechanisms and novel targets for immunotherapy. Genome Biol (2015) 16:64. doi: 10.1186/s13059-015-0620-6 25853550 PMC4377852

[5] Lee N Zakka LR Mihm MC Jr. Schatton T. Tumour-infiltrating lymphocytes in melanoma prognosis and cancer immunotherapy. Pathology (2016) 48:177–87. doi: 10.1016/j.pathol.2015.12.006 27020390

[6] Denkert C von Minckwitz G Darb-Esfahani S Lederer B Heppner BI Weber KE. Tumour-infiltrating lymphocytes and prognosis in different subtypes of breast cancer: a pooled analysis of 3771 patients treated with neoadjuvant therapy. Lancet Oncol (2018) 19:40–50. doi: 10.1016/S1470-2045(17)30904-X 29233559

[7] Galon J Bruni D. Approaches to treat immune hot, altered and cold tumours with combination immunotherapies. Nat Rev Drug Discovery (2019) 18:197–218. doi: 10.1038/s41573-018-0007-y 30610226

[8] Zhao Y Schaafsma E Gorlov IP Hernando E Thomas NE Shen R. A leukocyte infiltration score defined by a gene signature predicts melanoma patient prognosis. Mol Cancer Res (2019) 17:109–19. doi: 10.1158/1541-7786.MCR-18-0173 PMC6318018 30171176

[9] Morad G Helmink BA Sharma P Wargo JA. Hallmarks of response, resistance, and toxicity to immune checkpoint blockade. Cell (2021) 184:5309–37. doi: 10.1016/j.cell.2021.09.020 PMC8767569 34624224

[10] Domínguez Conde C Teichmann SA. Deciphering immunity at high plexity and resolution. Nat Rev Immunol (2020) 20:77–8. doi: 10.1038/s41577-019-0254-0 31797907

[11] Jia Q Chu H Jin Z Long H Zhu B. High-throughput single-сell sequencing in cancer research. Signal Transduct Target Ther (2022) 7:145. doi: 10.1038/s41392-022-00990-4 PMC9065032 35504878

[12] Ru B Wong CN Tong Y Zhong JY Zhong SSW Wu WC. TISIDB: an integrated repository portal for tumor-immune system interactions. Bioinformatics (2019) 35:4200–2. doi: 10.1093/bioinformatics/btz210 30903160

[13] Mootha VK Lindgren CM Eriksson KF Subramanian A Sihag S Lehar J. PGC-1alpha-responsive genes involved in oxidative phosphorylation are coordinately downregulated in human diabetes. Nat Genet (2003) 34:267–73. doi: 10.1038/ng1180 12808457

[14] Subramanian A Tamayo P Mootha VK Mukherjee S Ebert BL Gillette MA. Gene set enrichment analysis: a knowledge-based approach for interpreting genome-wide expression profiles. Proc Natl Acad Sci U.S.A. (2005) 102:15545–50. doi: 10.1073/pnas.0506580102 PMC1239896 16199517

[15] Foroutan M Bhuva DD Lyu R Horan K Cursons J Davis MJ. Single sample scoring of molecular phenotypes. BMC Bioinf (2018) 19:404. doi: 10.1186/s12859-018-2435-4 PMC6219008 30400809

[16] Andreopoulos B An A Wang X Schroeder M. A roadmap of clustering algorithms: finding a match for a biomedical application. Brief Bioinform (2009) 10:297–314. doi: 10.1093/bib/bbn058 19240124

[17] McCarthy DJ Chen Y Smyth GK. Differential expression analysis of multifactor RNA-Seq experiments with respect to biological variation. Nucleic Acids Res (2012) 40:4288–97. doi: 10.1093/nar/gks042 PMC3378882 22287627

[18] Love MI Huber W Anders S. Moderated estimation of fold change and dispersion for RNA-seq data with DESeq2. Genome Biol (2014) 15:550. doi: 10.1186/s13059-014-0550-8 25516281 PMC4302049

[19] Sherman BT Hao M Qiu J Jiao X Baseler MW Lane HC. DAVID: a web server for functional enrichment analysis and functional annotation of gene lists, (2021 update). Nucleic Acids Res (2022) 50:W216–221. doi: 10.1093/nar/gkac194 PMC9252805 35325185

[20] Griss J Viteri G Sidiropoulos K Nguyen V Fabregat A Hermjakob H. ReactomeGSA - efficient multi-omics comparative pathway analysis. Mol Cell Proteomics (2020) 19:2115–25. doi: 10.1074/mcp.TIR120.002155 PMC7710148 32907876

[21] Liberzon A Birger C Thorvaldsdóttir H Ghandi M Mesirov JP Tamayo P. The Molecular Signatures Database (MSigDB) hallmark gene set collection. Cell Syst (2015) 1:417–25. doi: 10.1016/j.cels.2015.12.004 PMC4707969 26771021

[22] Balachandran VP Gonen M Smith JJ DeMatteo RP. Nomograms in oncology: more than meets the eye. Lancet Oncol (2015) 16:e173–180. doi: 10.1016/S1470-2045(14)71116-7 PMC4465353 25846097

[23] Mayakonda A Lin DC Assenov Y Plass C Koeffler HP. Maftools: efficient and comprehensive analysis of somatic variants in cancer. Genome Res (2018) 28:1747–56. doi: 10.1101/gr.239244.118 PMC6211645 30341162

[24] Sharma P Hu-Lieskovan S Wargo JA Ribas A. Primary, adaptive, and acquired resistance to cancer immunotherapy. Cell (2017) 168:707–23. doi: 10.1016/j.cell.2017.01.017 PMC5391692 28187290

[25] Gajewski TF Schreiber H Fu YX. Innate and adaptive immune cells in the tumor microenvironment. Nat Immunol (2013) 14:1014–22. doi: 10.1038/ni.2703 PMC4118725 24048123

[26] Joyce JA Fearon DT. T cell exclusion, immune privilege, and the tumor microenvironment. Science (2015) 348:74–80. doi: 10.1126/science.aaa6204 25838376

[27] Spranger S Gajewski TF. Tumor-intrinsic oncogene pathways mediating immune avoidance. Oncoimmunology (2016) 5:e1086862. doi: 10.1080/2162402X.2015.1086862 27141343 PMC4839364

[28] Jiang P Gu S Pan D Fu J Sahu A Hu X. Signatures of T cell dysfunction and exclusion predict cancer immunotherapy response. Nat Med (2018) 24:1550–8. doi: 10.1038/s41591-018-0136-1 PMC6487502 30127393

[29] Ren D Hua Y Yu B Ye X He Z Li C. Predictive biomarkers and mechanisms underlying resistance to PD1/PD-L1 blockade cancer immunotherapy. Mol Cancer (2020) 19:19. doi: 10.1186/s12943-020-1144-6 32000802 PMC6993488

[30] Li T Fu J Zeng Z Cohen D Li J Chen Q. TIMER2.0 for analysis of tumor-infiltrating immune cells. Nucleic Acids Res (2020) 48:W509–w514. doi: 10.1093/nar/gkaa407 32442275 PMC7319575

[31] Newman AM Steen CB Liu CL Gentles AJ Chaudhuri AA Scherer F. Determining cell type abundance and expression from bulk tissues with digital cytometry. Nat Biotechnol (2019) 37:773–82. doi: 10.1038/s41587-019-0114-2 PMC6610714 31061481

[32] Merotto L,SG. immunedeconv: Methods for immune cell deconvolution (2022). Available at: https://github.com/omnideconv/immunedeconv.

[33] Li C Tang Z Zhang W Ye Z Liu F. GEPIA2021: integrating multiple deconvolution-based analysis into GEPIA. Nucleic Acids Res (2021) 49:W242–w246. doi: 10.1093/nar/gkab418 34050758 PMC8262695

[34] Wolf FA Angerer P Theis FJ. SCANPY: large-scale single-cell gene expression data analysis. Genome Biol (2018) 19:15. doi: 10.1186/s13059-017-1382-0 29409532 PMC5802054

[35] Ding ZB Shi YH Zhou J Qiu SJ Xu Y Dai Z. Association of autophagy defect with a malignant phenotype and poor prognosis of hepatocellular carcinoma. Cancer Res (2008) 68:9167–75. doi: 10.1158/0008-5472.CAN-08-1573 19010888

[36] Andersen JN Sathyanarayanan S Di Bacco A Chi A Zhang T Chen AH. Pathway-based identification of biomarkers for targeted therapeutics: personalized oncology with PI3K pathway inhibitors. Sci Transl Med (2010) 2:43ra55. doi: 10.1126/scitranslmed.3001065 20686178

[37] Yoshida A Tsuta K Wakai S Arai Y Asamura H Shibata T. Immunohistochemical detection of ROS1 is useful for identifying ROS1 rearrangements in lung cancers. Mod Pathol (2014) 27:711–20. doi: 10.1038/modpathol.2013.192 24186139

[38] Joseph JD Darimont B Zhou W Arrazate A Young A Ingalla E. The selective estrogen receptor downregulator GDC-0810 is efficacious in diverse models of ER+ breast cancer. Elife (2016) 5:1–33. doi: 10.7554/eLife.15828 PMC4961458 27410477

[39] Zhu GQ Tang Z Huang R Qu WF Fang Y Yang R. CD36(+) cancer-associated fibroblasts provide immunosuppressive microenvironment for hepatocellular carcinoma via secretion of macrophage migration inhibitory factor. Cell Discovery (2023) 9:25. doi: 10.1038/s41421-023-00529-z 36878933 PMC9988869

[40] MacDonald BT Tamai K He X. Wnt/beta-catenin signaling: components, mechanisms, and diseases. Dev Cell (2009) 17:9–26. doi: 10.1016/j.devcel.2009.06.016 19619488 PMC2861485

[41] Panera N Crudele A Romito I Gnani D Alisi A. Focal adhesion kinase: insight into molecular roles and functions in hepatocellular carcinoma. Int J Mol Sci (2017) 18:1–16. doi: 10.3390/ijms18010099 PMC5297733 28067792

[42] Yamaguchi H Hsu JM Yang WH Hung MC. Mechanisms regulating PD-L1 expression in cancers and associated opportunities for novel small-molecule therapeutics. Nat Rev Clin Oncol (2022) 19:287–305. doi: 10.1038/s41571-022-00601-9 35132224

[43] Cai G Freeman GJ. The CD160, BTLA, LIGHT/HVEM pathway: a bidirectional switch regulating T-cell activation. Immunol Rev (2009) 229:244–58. doi: 10.1111/j.1600-065X.2009.00783.x 19426226

[44] Tu TC Brown NK Kim TJ Wroblewska J Yang X Guo X. CD160 is essential for NK-mediated IFN-γ production. J Exp Med (2015) 212:415–29. doi: 10.1084/jem.20131601 PMC4354368 25711213

[45] Wein AN McMaster SR Takamura S Dunbar PR Cartwright EK Hayward SL. CXCR6 regulates localization of tissue-resident memory CD8 T cells to the airways. J Exp Med (2019) 216:2748–62. doi: 10.1084/jem.20181308 PMC6888981 31558615

[46] Heim TA Lin Z Steele MM Mudianto T Lund AW. CXCR6 promotes dermal CD8 (+) T cell survival and transition to long-term tissue residence. bioRxiv (2023) 1:33. doi: 10.1101/2023.02.14.528487 PMC12412903 40906897

[47] Riaz N Havel JJ Makarov V Desrichard A Urba WJ Sims JS. Tumor and microenvironment evolution during immunotherapy with nivolumab. Cell (2017) 171:934–949.e916. doi: 10.1016/j.cell.2017.09.028 29033130 PMC5685550

[48] Balar AV Galsky MD Rosenberg JE Powles T Petrylak DP Bellmunt J. Atezolizumab as first-line treatment in cisplatin-ineligible patients with locally advanced and metastatic urothelial carcinoma: a single-arm, multicentre, phase 2 trial. Lancet (2017) 389:67–76. doi: 10.1016/S0140-6736(16)32455-2 27939400 PMC5568632

[49] Ma L Hernandez MO Zhao Y Mehta M Tran B Kelly M. Tumor cell biodiversity drives microenvironmental reprogramming in liver cancer. Cancer Cell (2019) 36:418–430.e416. doi: 10.1016/j.ccell.2019.08.007 31588021 PMC6801104

[50] Sade-Feldman M Yizhak K Bjorgaard SL Ray JP de Boer CG Jenkins RW. Defining T cell states associated with response to checkpoint immunotherapy in melanoma. Cell (2018) 175:998–1013.e1020. doi: 10.1016/j.cell.2018.10.038 30388456 PMC6641984

[51] Yost KE Satpathy AT Wells DK Qi Y Wang C Kageyama R. Clonal replacement of tumor-specific T cells following PD-1 blockade. Nat Med (2019) 25:1251–9. doi: 10.1038/s41591-019-0522-3 PMC6689255 31359002

[52] Jerby-Arnon L Shah P Cuoco MS Rodman C Su MJ Melms JC. A cancer cell program promotes T cell exclusion and resistance to checkpoint blockade. Cell (2018) 175:984–997.e924. doi: 10.1016/j.cell.2018.09.006 30388455 PMC6410377

[53] Liu B Zhang Y Wang D Hu X Zhang Z. Single-cell meta-analyses reveal responses of tumor-reactive CXCL13(+) T cells to immune-checkpoint blockade. Nat Cancer (2022) 3:1123–36. doi: 10.1038/s43018-022-00433-7 36138134

[54] Xiao X Mo H Tu K. CTNNB1 mutation suppresses infiltration of immune cells in hepatocellular carcinoma through miRNA-mediated regulation of chemokine expression. Int Immunopharmacol (2020) 89:107043. doi: 10.1016/j.intimp.2020.107043 33039961

[55] Chen L Zhou Q Liu J Zhang W. CTNNB1 alternation is a potential biomarker for immunotherapy prognosis in patients with hepatocellular carcinoma. Front Immunol (2021) 12:759565. doi: 10.3389/fimmu.2021.759565 34777372 PMC8581472

[56] Guo Y Yang J Ren K Tian X Gao H Tian X. The heterogeneity of immune cell infiltration landscape and its immunotherapeutic implications in hepatocellular carcinoma. Front Immunol (2022) 13:861525. doi: 10.3389/fimmu.2022.861525 35355983 PMC8959995

[57] Giles RH van Es JH Clevers H. Caught up in a Wnt storm: Wnt signaling in cancer. Biochim Biophys Acta (2003) 1653:1–24. doi: 10.1016/S0304-419X(03)00005-2 12781368

[58] Cao X Liu B Cao W Zhang W Zhang F Zhao H. Autophagy inhibition enhances apigenin-induced apoptosis in human breast cancer cells. Chin J Cancer Res (2013) 25:212–22. doi: 10.3978/j.issn.1000-9604.2013.04.01 PMC3626985 23592903

[59] Oh JH Jang SJ Kim J Sohn I Lee JY Cho EJ. Spontaneous mutations in the single TTN gene represent high tumor mutation burden. NPJ Genom Med (2020) 5:33. doi: 10.1038/s41525-019-0107-6 PMC7424531 32821429

[60] Xue D Lin H Lin L Wei Q Yang S Chen X. TTN/TP53 mutation might act as the predictor for chemotherapy response in lung adenocarcinoma and lung squamous carcinoma patients. Transl Cancer Res (2021) 10:1284–94. doi: 10.21037/tcr-20-2568 PMC8798240 35116455

[61] Wang Z Wang C Lin S Yu X. Effect of TTN mutations on immune microenvironment and efficacy of immunotherapy in lung adenocarcinoma patients. Front Oncol (2021) 11:725292. doi: 10.3389/fonc.2021.725292 34513703 PMC8426356

[62] Yarchoan M Hopkins A Jaffee EM. Tumor mutational burden and response rate to PD-1 inhibition. N Engl J Med (2017) 377:2500–1. doi: 10.1056/NEJMc1713444 PMC6549688 29262275

[63] Braun DA Hou Y Bakouny Z Ficial M Sant' Angelo M Forman J. Interplay of somatic alterations and immune infiltration modulates response to PD-1 blockade in advanced clear cell renal cell carcinoma. Nat Med (2020) 26:909–18. doi: 10.1038/s41591-020-0839-y PMC7499153 32472114

[64] McGrail DJ Pilié PG Rashid NU Voorwerk L Slagter M Kok M. High tumor mutation burden fails to predict immune checkpoint blockade response across all cancer types. Ann Oncol (2021) 32:661–72. doi: 10.1016/j.annonc.2021.02.006 PMC8053682 33736924

[65] Oldham KA Parsonage G Bhatt RI Wallace DM Deshmukh N Chaudhri S. T lymphocyte recruitment into renal cell carcinoma tissue: a role for chemokine receptors CXCR3, CXCR6, CCR5, and CCR6. Eur Urol (2012) 61:385–94. doi: 10.1016/j.eururo.2011.10.035 22079021

[66] Karaki S Blanc C Tran T Galy-Fauroux I Mougel A Dransart E. CXCR6 deficiency impairs cancer vaccine efficacy and CD8(+) resident memory T-cell recruitment in head and neck and lung tumors. J Immunother Cancer (2021) 9:1–12. doi: 10.1136/jitc-2020-001948 PMC7949477 33692218

[67] Muthuswamy R McGray AR Battaglia S He W Miliotto A Eppolito C. CXCR6 by increasing retention of memory CD8(+) T cells in the ovarian tumor microenvironment promotes immunosurveillance and control of ovarian cancer. J Immunother Cancer (2021) 9:1–11. doi: 10.1101/2020.12.02.401729 PMC8491420 34607898

[68] Di Pilato M Kfuri-Rubens R Pruessmann JN Ozga AJ Messemaker M Cadilha BL. CXCR6 positions cytotoxic T cells to receive critical survival signals in the tumor microenvironment. Cell (2021) 184:4512–4530.e4522. doi: 10.1016/j.cell.2021.07.015 34343496 PMC8719451

[69] Wang B Wang Y Sun X Deng G Huang W Wu X. CXCR6 is required for antitumor efficacy of intratumoral CD8(+) T cell. J Immunother Cancer (2021) 9:1–13. doi: 10.1136/jitc-2021-003100 PMC8407215 34462326

[70] Mossanen JC Kohlhepp M Wehr A Krenkel O Liepelt A Roeth AA. CXCR6 inhibits hepatocarcinogenesis by promoting natural killer T- and CD4(+) T-cell-dependent control of senescence. Gastroenterology (2019) 156:1877–1889.e1874. doi: 10.1053/j.gastro.2019.01.247 30710528

[71] Zheng L Qin S Si W Wang A Xing B Gao R. Pan-cancer single-cell landscape of tumor-infiltrating T cells. Science (2021) 374:abe6474. doi: 10.1126/science.abe6474 34914499

[72] Duhen T Duhen R Montler R Moses J Moudgil T de Miranda NF. Co-expression of CD39 and CD103 identifies tumor-reactive CD8 T cells in human solid tumors. Nat Commun (2018) 9:2724. doi: 10.1038/s41467-018-05072-0 30006565 PMC6045647

[73] Thommen DS Koelzer VH Herzig P Roller A Trefny M Dimeloe S. A transcriptionally and functionally distinct PD-1(+) CD8(+) T cell pool with predictive potential in non-small-cell lung cancer treated with PD-1 blockade. Nat Med (2018) 24:994–1004. doi: 10.1038/s41591-018-0057-z 29892065 PMC6110381

[74] Caushi JX Zhang J Ji Z Vaghasia A Zhang B Hsiue EH. Transcriptional programs of neoantigen-specific TIL in anti-PD-1-treated lung cancers. Nature (2021) 596:126–32. doi: 10.1038/s41586-021-03752-4 PMC8338555 34290408

[75] Liu B Hu X Feng K Gao R Xue Z Zhang S. Temporal single-cell tracing reveals clonal revival and expansion of precursor exhausted T cells during anti-PD-1 therapy in lung cancer. Nat Cancer (2022) 3:108–21. doi: 10.1038/s43018-021-00292-8 35121991

