# Supplementary material for: Integrated single cell and bulk sequencing analysis identifies tumor reactive CXCR6+ CD8 T cells as a predictor of immune infiltration and immunotherapy outcomes in hepatocellular carcinoma
Source: Front Oncol. 2023 Aug 1;13:1099385. doi: 10.3389/fonc.2023.1099385 (PMC10430781; doi:10.3389/fonc.2023.1099385)
Supplement: Supplementary file 6 [file Image_1.pdf]

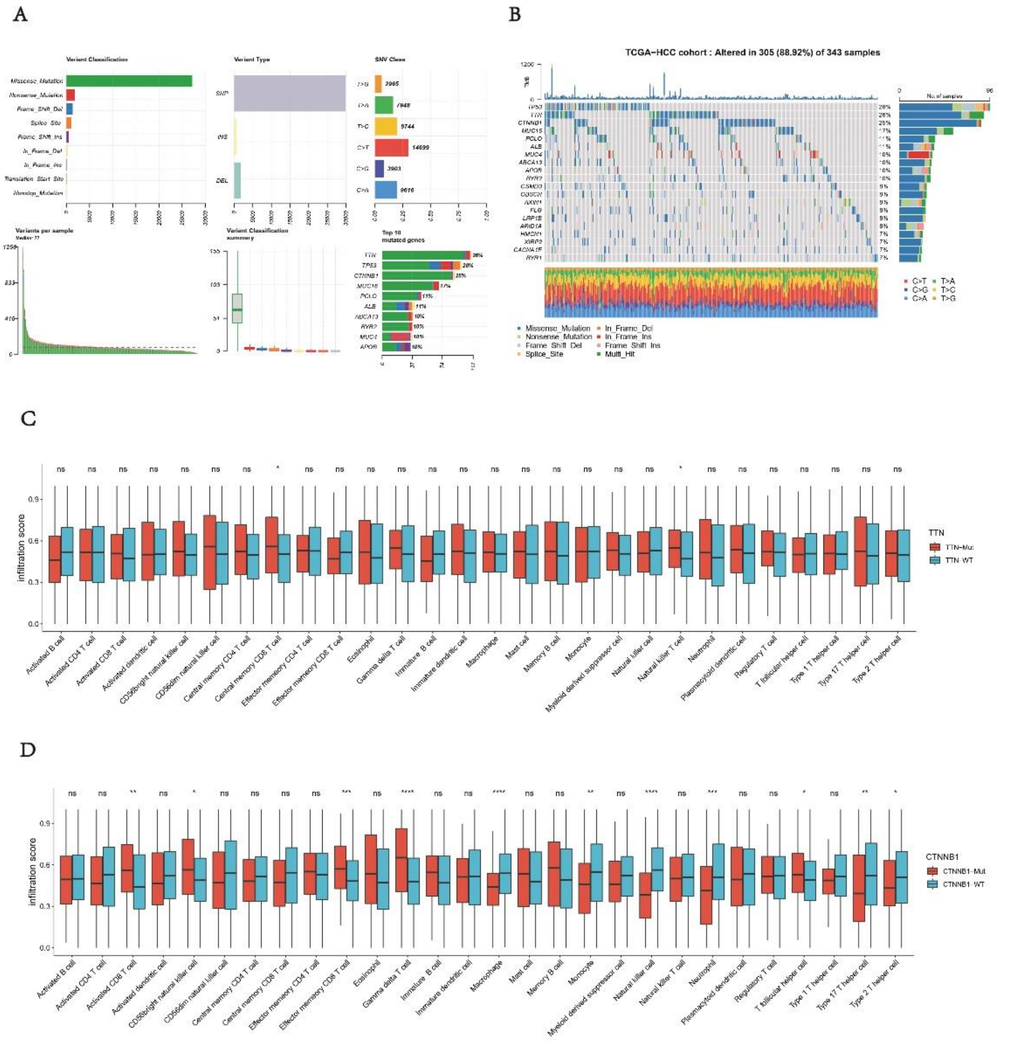

Fig. S1 : Gene mutations analysis of TCGA-HCC cohort

(A) Gene mutation summary of TCGA HCC cohort

(B) Waterfall plot shows the top mutations of TCGA HCC cohort

(C) Infiltration score of different immune cell types in TTN mutation group and wild group. *P<0.05; **P<0.01; ***P<0.001; and ****P<0.0001.

(D) Infiltration score of different immune cell types in TTN mutation group and wild group. *P<0.05; **P<0.01; ***P<0.001; and ****P<0.0001.

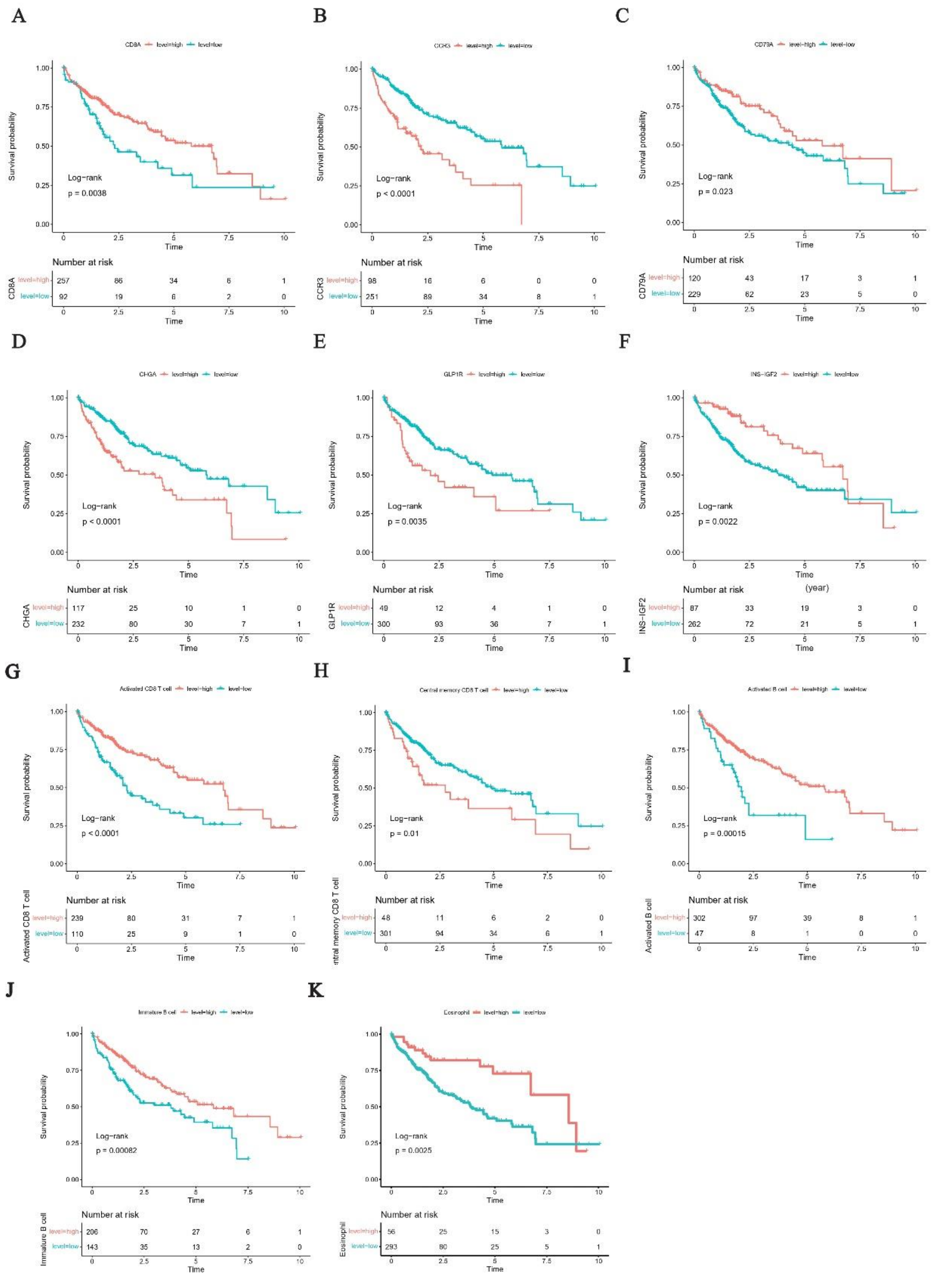

Fig. S2 : Survival curve of risk genes and risk cell types screened by univariate cox regression and lasso regression analysis.

(A-F) Survival curve of CD8A, CCR3, CD79A, CHGA, GLPAR, INS-IGF2.

(G-K) Survival curve of activated CD8 T cell, effector memory CD8 T cell, activated B cell, immune B cell and eosinophil.

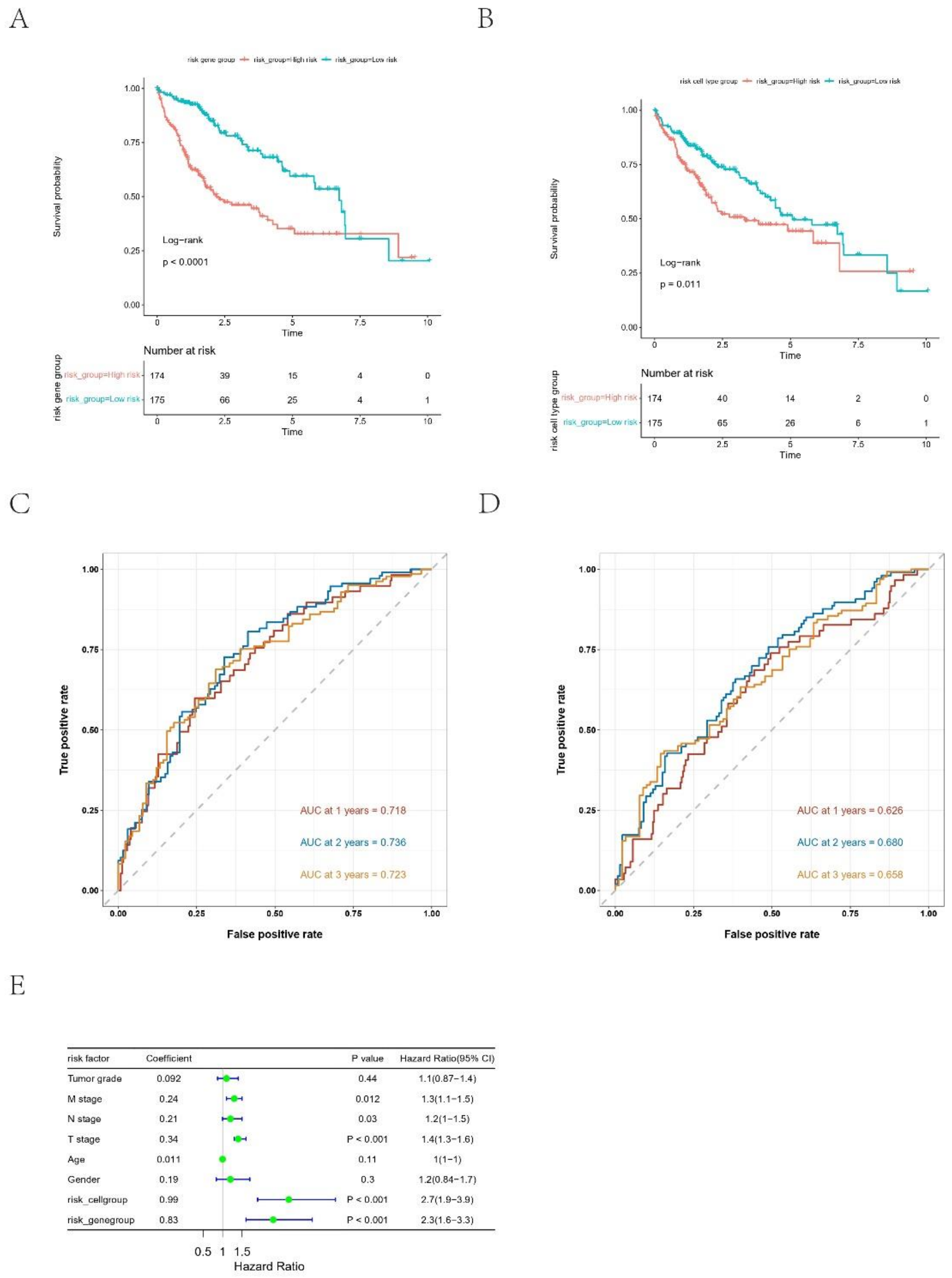

Fig. S3 : construction and validation of prognostic model based on risk genes and risk cell types

(A) survival curve of high and low risk gene score group.
(B) survival curve of high and low risk cell type score group.
(C) ROC curve of high and low risk gene score group for predicting patients' survival
(D) ROC curve of high and low risk cell type score group for predicting patients' survival
(E) Univariate cox regression analysis of risk gene score, risk cell type score and clinicopathologic factors

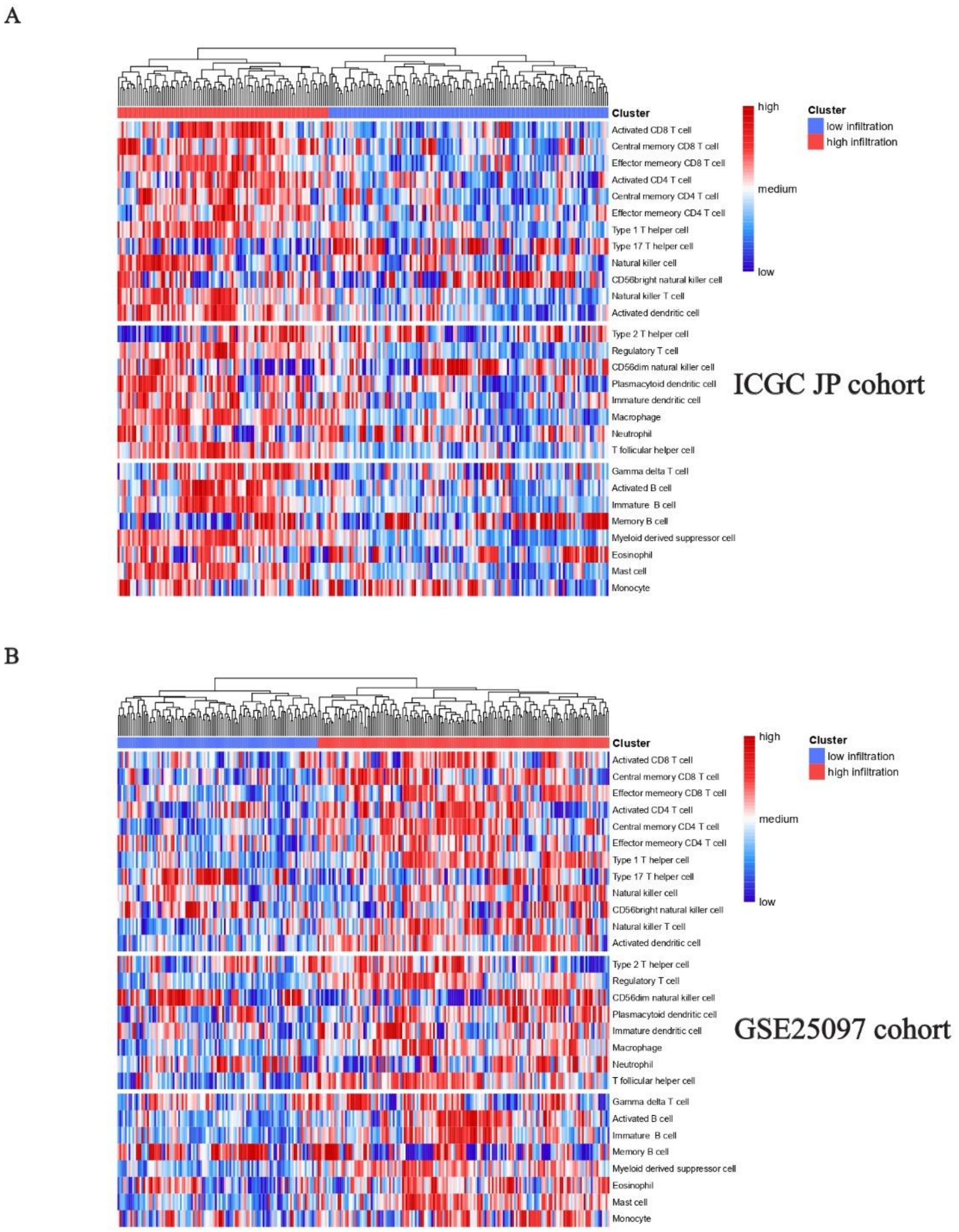

Fig. S4 : high and low infiltration clustering of ICGC JP cohort and GSE25097 cohort.

(A) Heatmap show clustering of ICGC JP cohort into high and low infiltration group.

(B) Heatmap show clustering of GSE25097 cohort into high and low infiltration group.

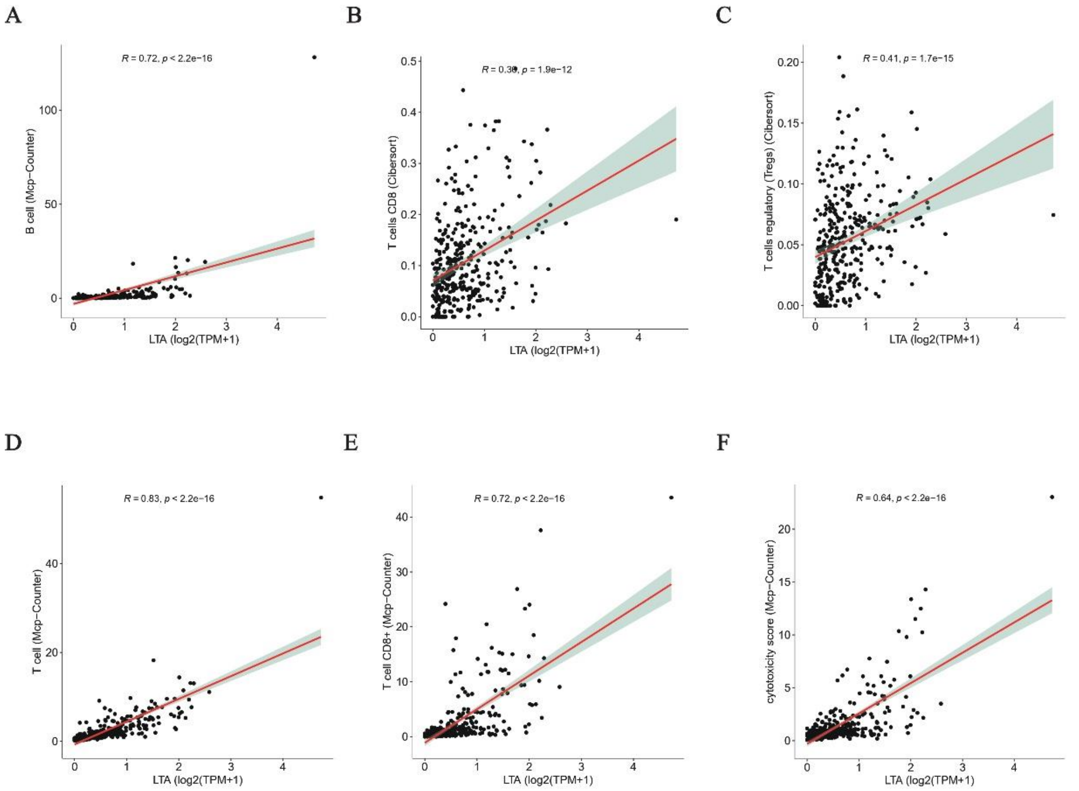

Fig. S5 : correlation analysis of LTA and B cell infiltration score and T cell infiltration score, calculated by CIBERSORT and MCP-COUNTER.

(A) Correlation analysis of LTA and B cell infiltration score (MCP-COUNTER).
(B) Correlation analysis of LTA and CD8 T cell infiltration score (CIBERSORT).
(C) Correlation analysis of LTA and regulatory T cell infiltration score (CIBERSORT).
(D) Correlation analysis of LTA and T cell infiltration score (MCP-COUNTER).
(E) Correlation analysis of LTA and CD8 T cell infiltration score (MCP-COUNTER).
(F) Correlation analysis of LTA and cytotoxicity score (MCP-COUNTER).

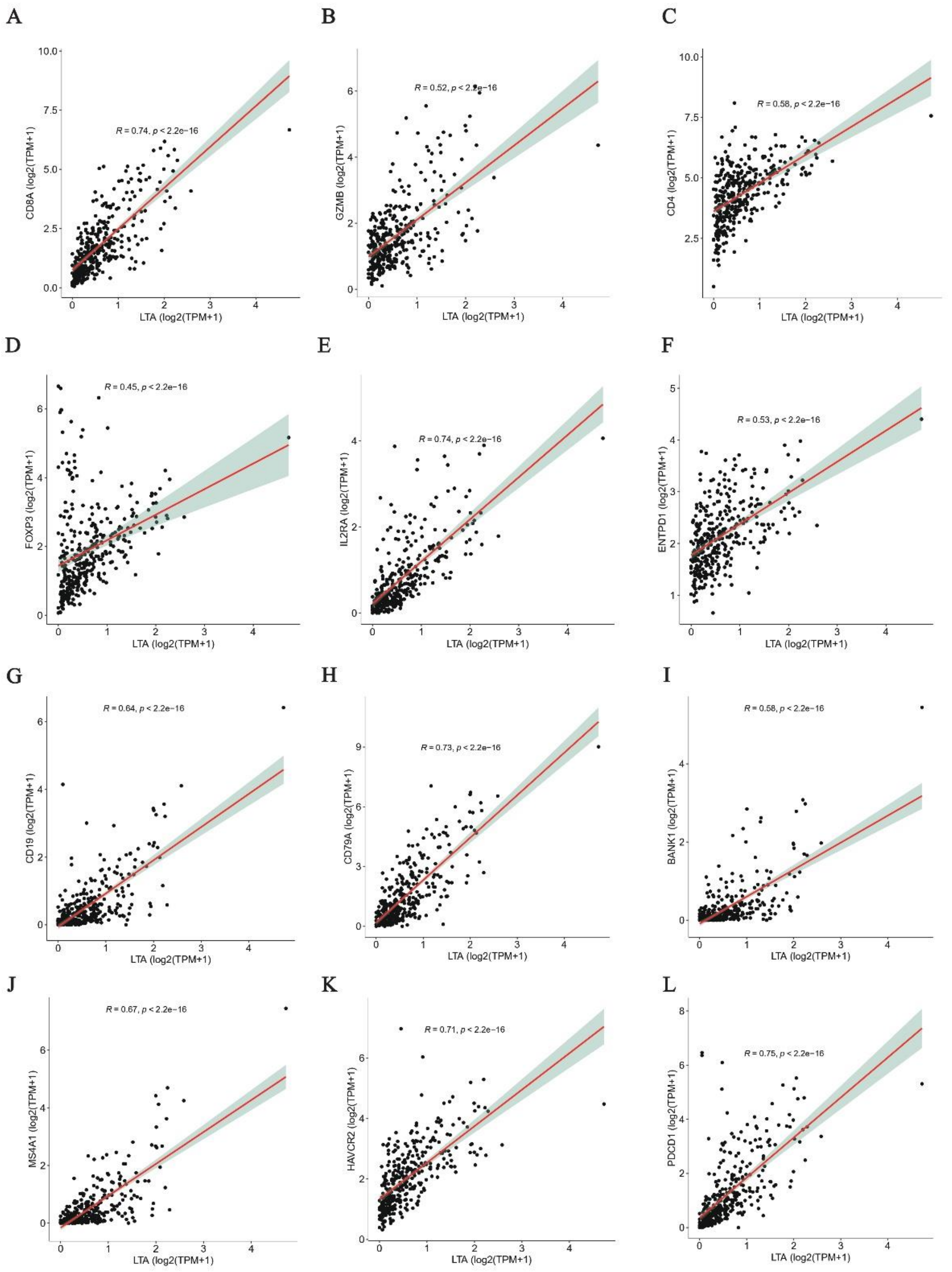

Fig. S6 : Correlation analysis of LTA and T cell markers (CD8A, GZMB, CD4, FOXP3, IL2RA, ENTPD1) , B cell markers (CD19, CD79A, BANK1, MS4A1) and immune checkpoints molecules (HAVCR2, PDCD1)

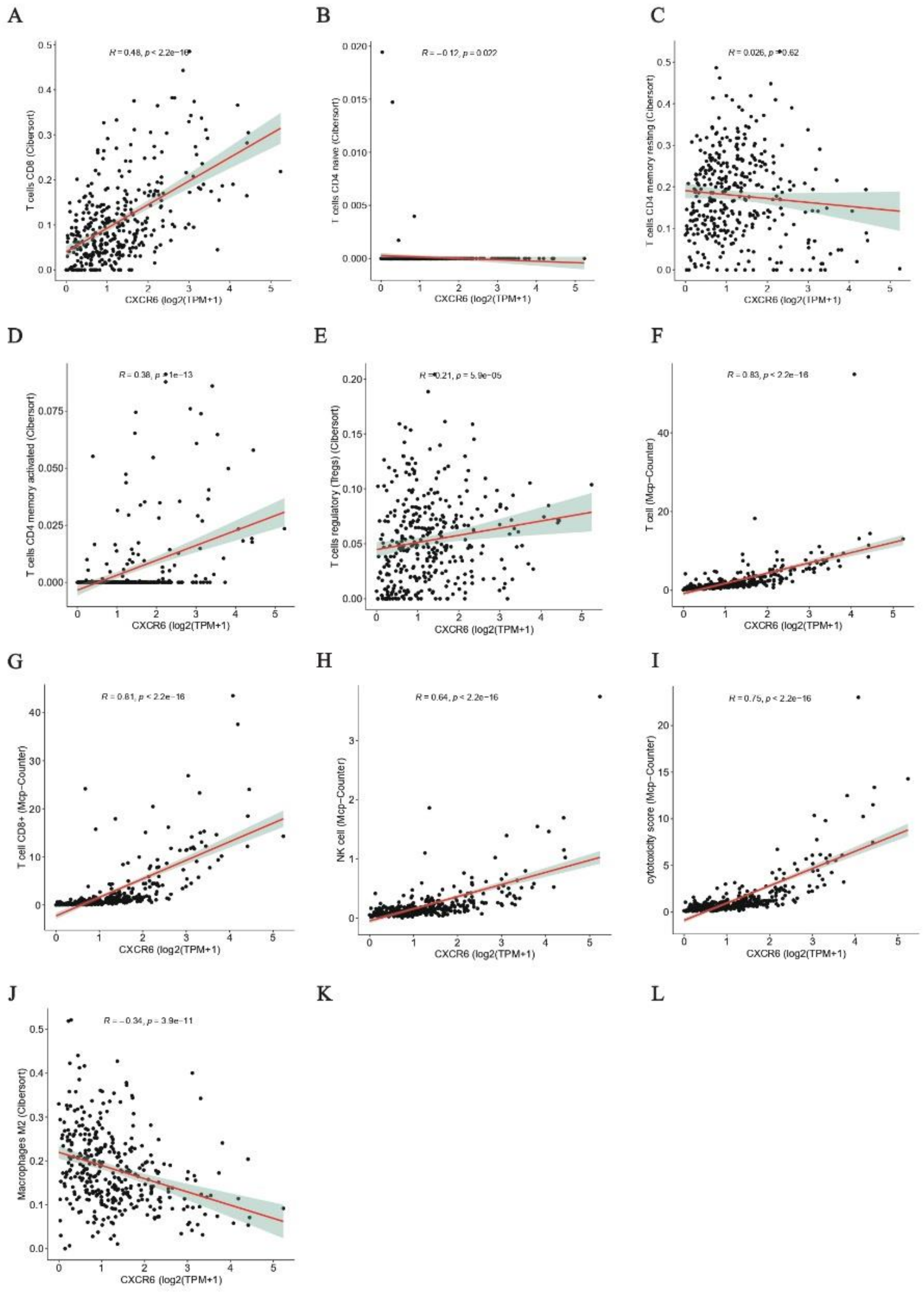

Fig. S7 : Correlation analysis of CXCR6 and T cell , NK cell and M2 type macrophage infiltration score, calculated by CIBERSORT and MCP-COUNTER.

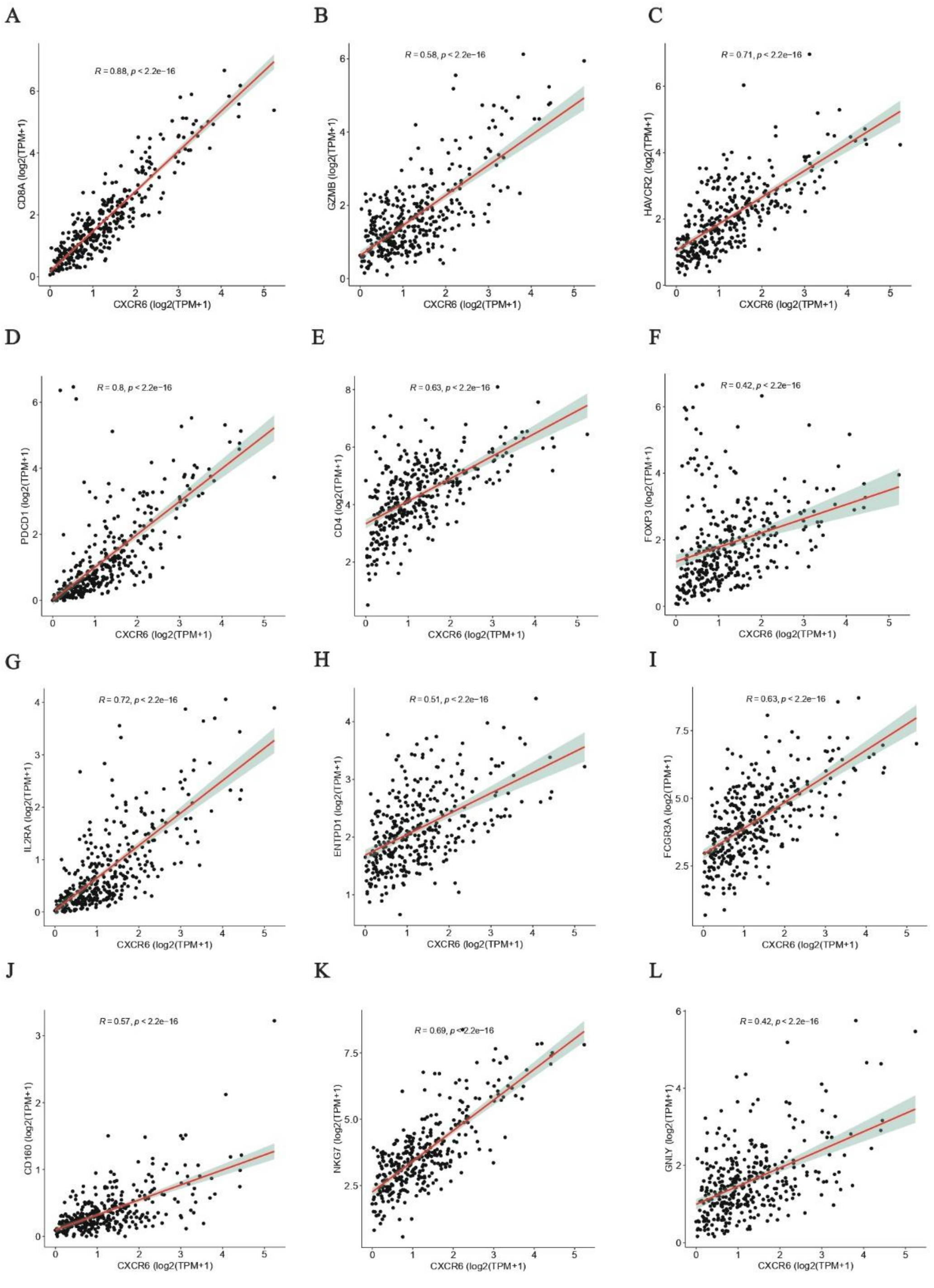

Fig. S8 : Correlation analysis of CXCR6 and T cell markers (CD8A, GZMB, CD4, FOXP3, IL2RA, ENTPD1) , NK cell markers (FCGR3A, CD160, NKG7 and GNLY) and immune checkpoints molecules (HAVCR2, PDCD1)

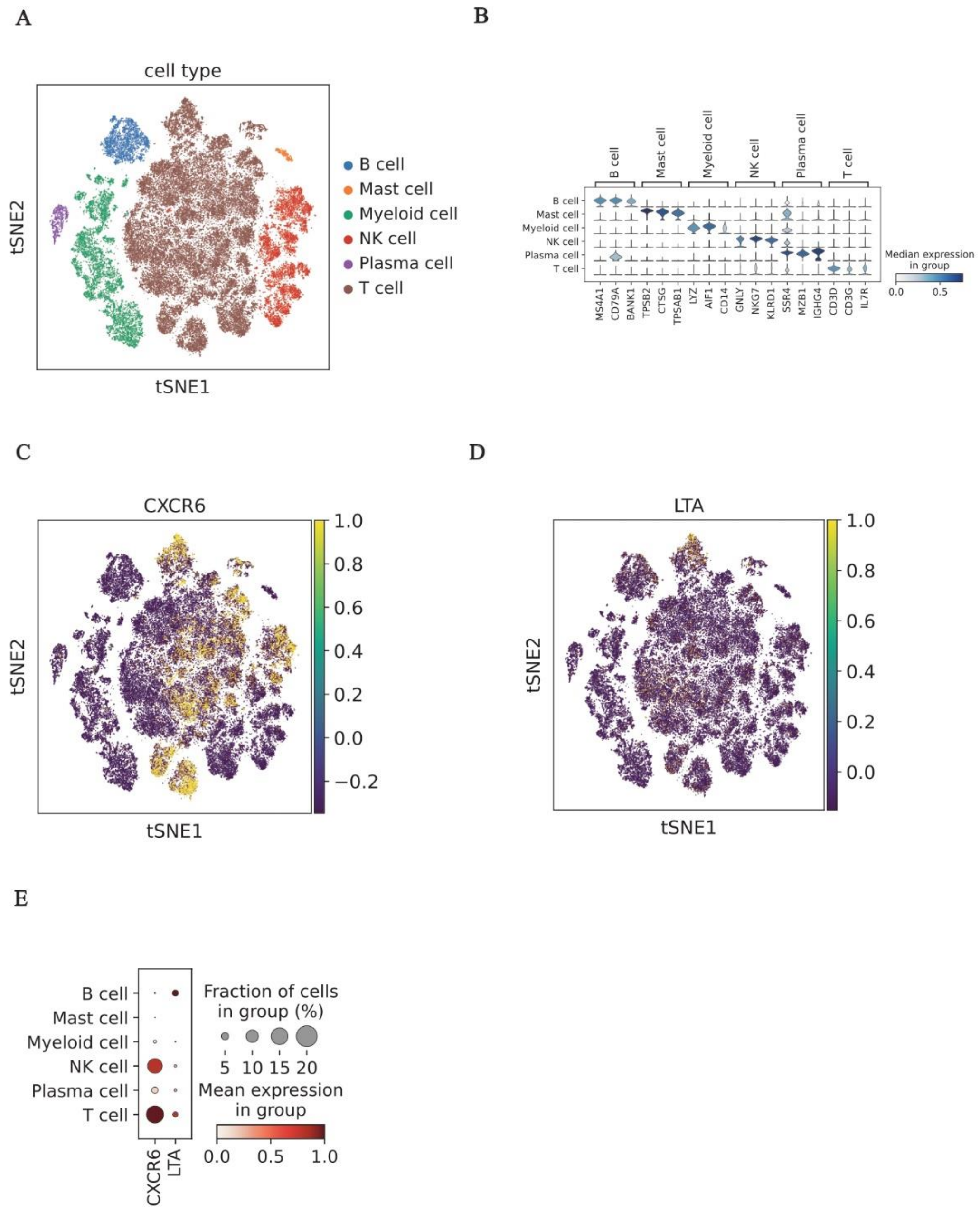

Fig. S9 : Expression validation of CXCR6 and LTA in GSE140228 dataset

(A) Cell type annotation of GSE140228 dataset, visualized by TSNE
(B) Cell type markers of six main cell types visualized by stacked violin plot
(C) Feature plot of CXCR6 in global TSNE
(D) Feature plot of LTA in global TSNE
(E) Dot plot show the expression pattern of CXCR6 and LTA

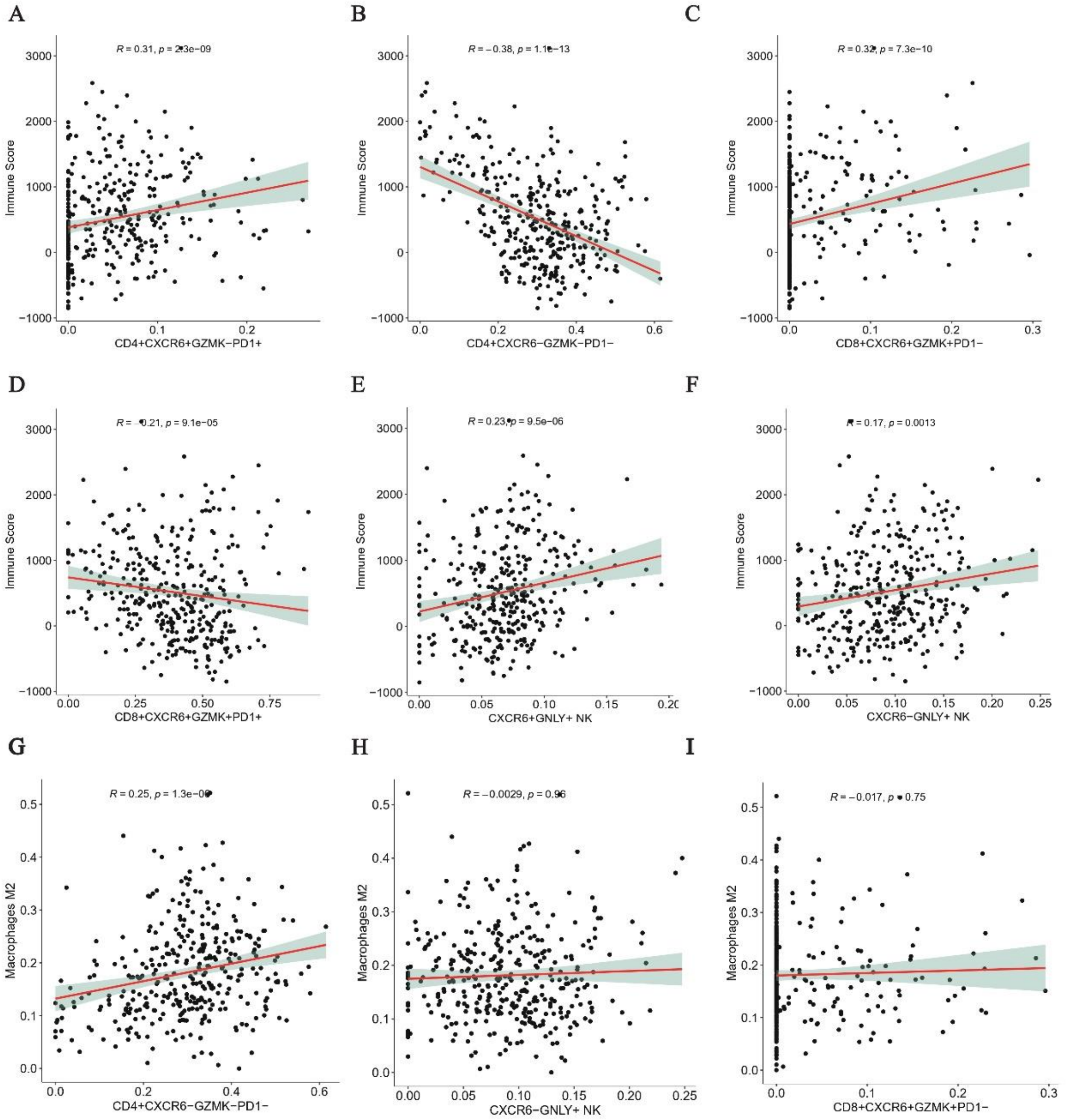

Fig. S10: Correlation analysis of different T/NK cell subtype infiltration score and immune score (calculated by ESTIMATE algorithm), M2 type macrophage infiltration score (calculated by CIBERSORT). The infiltration score of different T cell subtype are deconvoluted by CIBERSORT. T/NK cell subtypes are clustered according to relative expression level of CXCR6, GZMK, PDCD1 and GNLY.

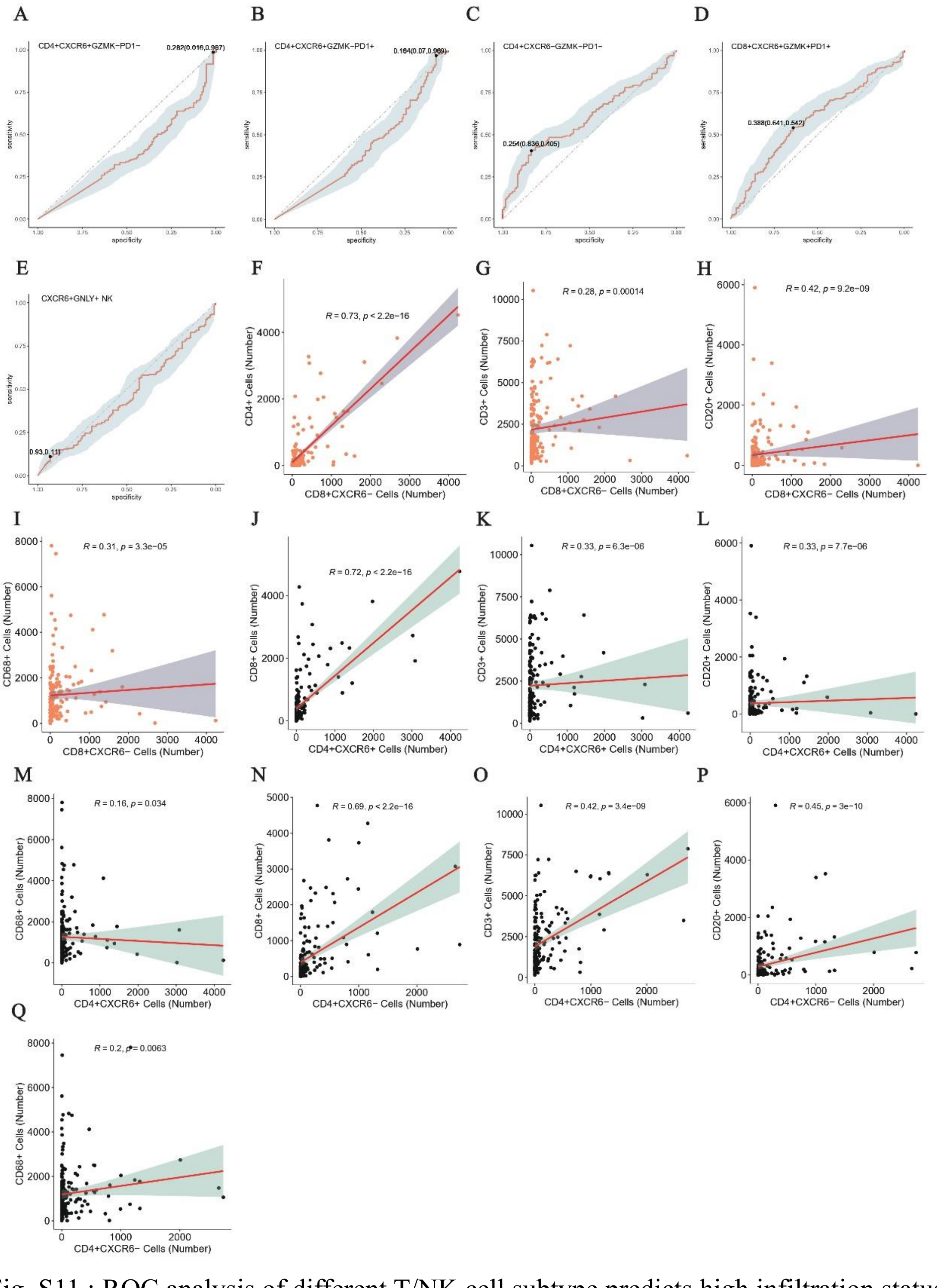

Fig. S11 : ROC analysis of different T/NK cell subtype predicts high infiltration status in TCGA HCC cohort and correlation analysis of CXCR6^{+} T cell (our HCC TMA cohort) with immune cell infiltration markers (CD3, CD4, CD8, CD20, CD68).

(A-E) ROC showed the sensitivity and specificity of T and NK subtypes in predicting high infiltration status, respectively. The infiltration score of different T cell subtype are deconvoluted by CIBERSORT. T/NK cell subtypes are clustered according to relative expression level of CXCR6, GZMK, PDCD1 and GNLY.

(F-I) Correlation analysis between CD8^{+}CXCR6^{-} T cells with CD3, CD4, CD20,

CD68.

(J-M) Correlation analysis between CD4^{+}CXCR6^{+} T cells with CD3, CD8, CD20, CD68.

(N-Q) Correlation analysis between CD4^{+}CXCR6^{-} T cells with CD3, CD8, CD20, CD68.

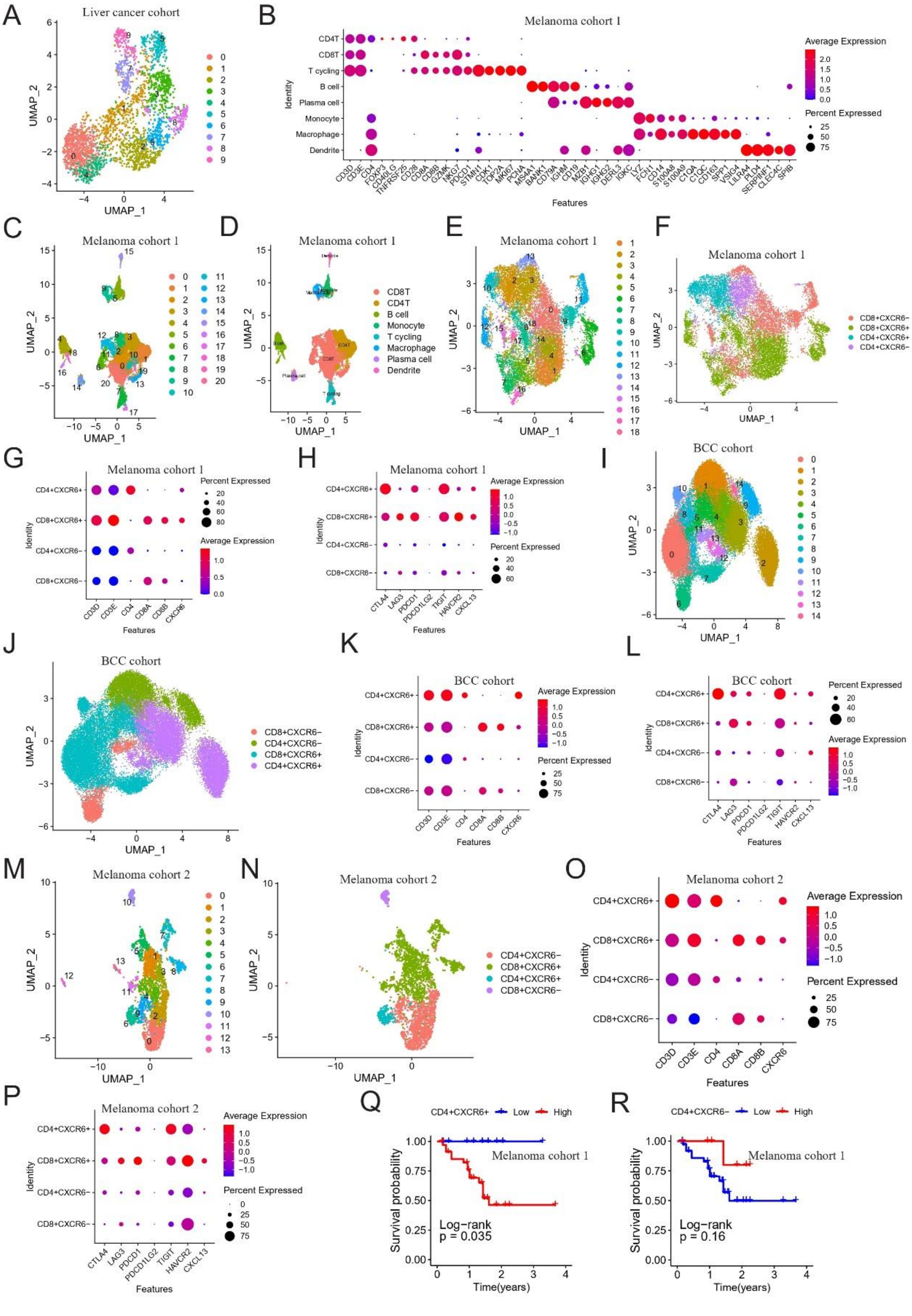

Fig. S12 : Clustering and cell annotation of four single-cell sequencing immunotherapy cohort

(A) UMAP plots showing the clustering of T cells from liver cancer cohort (GSE125449). Cells were colored by Seurat clusters.

(B) Dot plot showed cell markers of main cell types in melanoma cohort 1 (GSE120575).

(C) UMAP plots showing the clustering of all cells from melanoma cohort 1. Cells were colored by Seurat clusters.

(D) UMAP plots showing the cell types from melanoma cohort 1. Cells were colored by cell type.

(E) UMAP plots showing the clustering of T cells from melanoma cohort 1. Cells were colored by Seurat clusters.

(F) UMAP plots showing the T cell types from melanoma cohort 1. Cells were colored by cell type. T cell annotation are according to the relative expression level of CD4, CD8A, CD8B, CXCR6.

(G) Dot plot showed cell markers expression of T cell types in melanoma cohort 1.

(H) Expression level of immune checkpoint molecules in T cell subtypes from melanoma cohort 1.

(I) UMAP plots showing the clustering of T cells from BCC cohort. Cells were colored by Seurat clusters.

(J) UMAP plots showing the T cell types from BCC cohort. Cells were colored by cell type. T cell annotation are according to the relative expression level of CD4, CD8A, CD8B, CXCR6.

(K) Dot plot showed cell markers expression of T cell types in BCC cohort.

(L) Expression level of immune checkpoint molecules in T cell subtypes from BCC cohort.

(M) UMAP plots showing the clustering of T cells from melanoma cohort 2. Cells were colored by Seurat clusters.

(N) UMAP plots showing the T cell types from melanoma cohort 2. Cells were colored by cell type. T cell annotation are according to the relative expression level of CD4, CD8A, CD8B, CXCR6.

(O) Dot plot showed cell markers expression of T cell types in melanoma cohort 2.

(P) Expression level of immune checkpoint molecules in T cell subtypes from melanoma cohort 2.

(Q) Kaplan-Meier survival analysis of $CD4^+CXCR6^+$ and $CD4^+CXCR6^-$ in melanoma cohort 1. Log-rank test was used for statistical analysis. Left panel represented $CD4^+CXCR6^+$ and right panel represented $CD4^+CXCR6^-$.
